# Patterns of Neural Network Functional Connectivity Associated With Mania/Hypomania and Depression Risk in 3 Independent Young Adult Samples

**DOI:** 10.1001/jamapsychiatry.2023.4150

**Published:** 2023-11-01

**Authors:** Maya C. Schumer, Michele A. Bertocci, Haris A. Aslam, Simona Graur, Genna Bebko, Richelle S. Stiffler, Alexander S. Skeba, Tyler J. Brady, Osasumwen E. Benjamin, Yiming Wang, Henry W. Chase, Mary L. Phillips

**Affiliations:** 1 Department of Psychiatry, University of Pittsburgh School of Medicine, Pittsburgh, Pennsylvania

## Abstract

**Importance:**

Mania/hypomania is the pathognomonic feature of bipolar disorder (BD). Established, reliable neural markers denoting mania/hypomania risk to help with early risk detection and diagnosis and guide the targeting of pathophysiologically informed interventions are lacking.

**Objective:**

To identify patterns of neural responses associated with lifetime mania/hypomania risk, the specificity of such neural responses to mania/hypomania risk vs depression risk, and the extent of replication of findings in 2 independent test samples.

**Design, Setting, and Participants:**

This cross-sectional study included 3 independent samples of young adults aged 18 to 30 years without BD or active substance use disorder within the past 3 months who were recruited from the community through advertising. Of 603 approached, 299 were ultimately included and underwent functional magnetic resonance imaging at the University of Pittsburgh, Pittsburgh, Pennsylvania, from July 2014 to May 2023.

**Main Outcomes and Measures:**

Activity and functional connectivity to approach-related emotions were examined using a region-of-interest mask supporting emotion processing and emotional regulation. The Mood Spectrum Self-Report assessed lifetime mania/hypomania risk and depression risk. In the discovery sample, elastic net regression models identified neural variables associated with mania/hypomania and depression risk; multivariable regression models identified the extent to which selected variables were significantly associated with each risk measure. Multivariable regression models then determined whether associations in the discovery sample replicated in both test samples.

**Results:**

A total of 299 participants were included. The discovery sample included 114 individuals (mean [SD] age, 21.60 [1.91] years; 80 female and 34 male); test sample 1, 103 individuals (mean [SD] age, 21.57 [2.09] years; 30 male and 73 female); and test sample 2, 82 individuals (mean [SD] age, 23.43 [2.86] years; 48 female, 29 male, and 5 nonbinary). Associations between neuroimaging variables and Mood Spectrum Self-Report measures were consistent across all 3 samples. Bilateral amygdala–left amygdala functional connectivity and bilateral ventrolateral prefrontal cortex–right dorsolateral prefrontal cortex functional connectivity were positively associated with mania/hypomania risk: discovery omnibus χ^2^ = 1671.7 (*P* < .001); test sample 1 omnibus χ^2^ = 1790.6 (*P* < .001); test sample 2 omnibus χ^2^ = 632.7 (*P* < .001). Bilateral amygdala–left amygdala functional connectivity and right caudate activity were positively associated and negatively associated with depression risk, respectively: discovery omnibus χ^2^ = 2566.2 (*P* < .001); test sample 1 omnibus χ^2^ = 2935.9 (*P* < .001); test sample 2 omnibus χ^2^ = 1004.5 (*P* < .001).

**Conclusions and Relevance:**

In this study of young adults, greater interamygdala functional connectivity was associated with greater risk of both mania/hypomania and depression. By contrast, greater functional connectivity between ventral attention or salience and central executive networks and greater caudate deactivation were reliably associated with greater risk of mania/hypomania and depression, respectively. These replicated findings indicate promising neural markers distinguishing mania/hypomania–specific risk from depression-specific risk and may provide neural targets to guide and monitor interventions for mania/hypomania and depression in at-risk individuals.

## Key Points

**Question:**

Are there objective, reproducible neural markers that can distinguish mania/hypomania from depression risk?

**Findings:**

In 3 independent samples comprising 299 young adults, neural response patterns differentially associated with mania/hypomania and depression risk were identified and replicated. Greater bilateral amygdala–left amygdala functional connectivity was associated with greater mania/hypomania and depression risk, and greater bilateral ventrolateral prefrontal cortex–right dorsolateral prefrontal cortex functional connectivity and greater right caudate deactivation were associated with greater mania/hypomania and depression risk, respectively.

**Meaning:**

Neural markers reliably associated with mania/hypomania and depression risk may help identify young adults at risk of bipolar disorder and provide treatment targets for early interventions.

## Teaser

This cross-sectional study evaluates neural responses associated with risk of mania/hypomania and depression in 3 independent test samples.

## Introduction

Bipolar disorder (BD), the pathognomonic feature of which is mania/hypomania,^1^ has peak onset in early adulthood.^2^ BD is often difficult to accurately diagnose, due to misreporting mania/hypomania, resulting in its frequent misclassification as unipolar depression.^3^ To facilitate earlier and more accurate BD diagnoses, there is a vital need for objective markers distinguishing risk of mania/hypomania from risk of depression.^1^ Identifying neural markers associated with risk for these symptoms can provide objective markers reflecting underlying pathophysiological processes and neural targets to guide and monitor interventions for BD.^4^

Three methodological approaches can be used to identify neural markers denoting risk of mania/hypomania. First, neuroimaging paradigms assessing neural responses underlying attention to approach-related emotional cues are especially useful for identifying markers associated with risk of mania/hypomania^4^ because of previous work highlighting behavioral approach system overactivity^5,6^ in mania/hypomania, resulting in attentional predisposition toward positive emotional stimuli^7^ (eg, happy faces and rewards^8^); emotional dysregulation^9^; and heightened positive affectivity, irritability, and anger.^10^

Second, studies should examine associations among neural responses in these contexts and comprehensive measures of mania/hypomania and depression risk. The Mood Spectrum Self-Report (MOODS-SR)^11^ measures lifetime vulnerability to mania/hypomania and depression, detecting subthreshold-level and threshold-level manifestations, with manic and depressive mood domain scales assessing the subtle behaviors over an individual’s lifetime that are associated with mania/hypomania and/or depression risk. By contrast, standard clinical rating scales assess current mania/hypomania or depression severity.^11,12,13^ The manic mood domain in particular discriminates between individuals with BD vs unipolar depression,^11,13,14^ highlighting the potential of this scale to screen for mania/hypomania and thus BD-specific risk in young adults on the BD spectrum, some of whom might already be diagnosed with unipolar depression.^15,16^

Additionally, studies should examine large-scale neural networks relevant to emotional dysregulation in approach-related contexts, including the central executive network (CEN), default mode network (DMN), salience network (SN), and ventral attention network (VAN).^8,17,18^ The CEN, centered on the dorsolateral prefrontal cortex (dlPFC) and caudate, supports attention-demanding tasks, decision making, and maintaining cognitive and emotional control.^17,19^ The DMN, centered on the medial prefrontal cortex (mPFC), precuneus, and posterior cingulate cortex, supports self-referential processing and introspection.^20^ The SN, centered on the (caudal) ventrolateral prefrontal cortex (vlPFC),^21^ dorsal anterior cingulate cortex (dACC), anterior insula, and amygdala, monitors, identifies, and integrates emotionally salient information^19^ with connections to reward (ventral striatum) and motor learning (putamen)^22^ regions. The VAN, centered on the (rostral) vlPFC and anterior insula, orients attention to emotionally salient stimuli and coactivates with CEN regions (eg, the dlPFC) when external stimuli refocus attention.^23^ Additionally, the orbitofrontal cortex (OFC) supports stimulus value encoding and updating,^24^ important for emotionally salient information processing.

Previous neuroimaging studies in individuals with BD reported amygdala and vlPFC hyperactivity to emotional stimuli,^8,25^ CEN hypoactivity^26^ and reduced CEN-amygdala functional connectivity during different cognitive and emotional-regulation paradigms^26^; SN hyperactivity to potential reward cues, notably in left caudal vlPFC^8,27,28,29^; and VAN or CEN^18,30,31^ and SN^32^ hyperconnectivity and altered DMN functional connectivity^33,34^ during resting state. Amygdala hyperactivity to positive emotional stimuli^25^; caudal left vlPFC hyperactivity to potential reward cues^27^; and elevated CEN, SN, and VAN functional connectivity in different contexts^30,31,32^ also distinguished individuals with BD from those with unipolar depression. Similarly, in a meta-analysis^35^ of functional neuroimaging studies in adult BD, we identified robust, reproducible, condition-dependent neural patterns characterizing BD, including altered left amygdala activity across emotional tasks, altered CEN activity across cognitive tasks, altered DMN resting-state functional connectivity, and altered CEN right caudate activity across all task types.

To our knowledge, no studies to date have combined all 3 of the above approaches. The few studies assessing neural markers of mania/hypomania risk in young adults reported similar findings of reward-related striatal and SN (insula) and emotional regulation–related amygdala hyperactivity in individuals who were hypomania prone vs control individuals^36,37^; positive associations between mania/hypomania risk (measured by the MOODS-SR) and SN activity during reward processing^38,39^; and reduced resting-state functional connectivity between the ventral striatum and CEN regions in individuals at highest risk for BD.^15^ Studies in familial at-risk populations (eg, offspring of individuals affected by BD) and youth affected by BD similarly reported altered amygdala, striatal, CEN, and VAN or SN activity and altered amygdala-VAN and amygdala-SN functional connectivity during emotion processing and emotional regulation.^40,41,42,43^ Yet, although childhood-onset BD is a more severe form of BD,^44^ it is less common than adult-onset BD.^45^ Thus, there remains a critical need for studies to examine young adults at risk of mania/hypomania.^35^ There is also a need to address the larger replication crisis in brain-behavior research^46^ and replicate neuroimaging findings in independent samples^47,48^ to yield reliable, robust neural markers of BD risk.^38^

The goal of the present study was to identify neural markers of mania/hypomania vs depression risk in young adults. We first aimed to identify neural markers of mania/hypomania risk during approach-related emotion processing using the MOODS-SR manic mood measure of mania/hypomania risk in a discovery sample of young adults aged 18 to 30 years recruited from the general community across a range of such risk. Based on the previous findings described above, we hypothesized that greater mania/hypomania risk would be associated with amygdala and SN hyperactivity, CEN hypoactivity, CEN-amygdala hypoconnectivity, altered DMN functional connectivity, and VAN-CEN hyperconnectivity to approach-related emotional cues (hypothesis 1). We next aimed to determine whether associations found were specific to mania/hypomania risk. We hypothesized that findings would be mania/hypomania specific, and not common to depression risk (hypothesis 2). We lastly aimed to determine whether risk-specific associations could be independently replicated. We hypothesized that these associations would replicate in 2 independent young adult test samples (hypothesis 3).

## Methods

### Participants and Measures

A total of 299 young adult participants composed 3 independent samples recruited across a range of subsyndromal-syndromal affective and anxiety psychopathology (excluding BD and active substance use disorder within the past 3 months). Participants were recruited through student counseling centers, participant registries, and community advertisements. The University of Pittsburgh institutional review board approved this study; all participants gave written informed consent. For additional exclusion criteria, medication, and power calculations, see the eMethods in Supplement 1.

The discovery sample included 114 individuals (mean [SD] age, 21.60 [1.91] years; 80 female and 34 male; 36 with lifetime diagnoses of major depressive disorder, attention-deficit/hyperactivity disorder, or anxiety disorders and 78 without). Test sample 1 included 103 individuals (mean [SD] age, 21.57 [2.09] years; 30 male and 73 female; 47 with psychiatric diagnoses and 56 without). Test sample 2 included 82 individuals (mean [SD] age, 23.43 [2.86] years; 48 female, 29 male, and 5 nonbinary; 19 with psychiatric diagnoses and 63 without) (Table 1).

**Table 1.** Demographic and Clinical Measures of the 3 Samples

Demographic and clinical variables^a^	Discovery sample (n = 114)	Test sample 1 (n = 103)	Test sample 2 (n = 82)	Statistic	*P* value
Age, mean (SD), y	21.60 (1.91)	21.57 (2.09)	23.43 (2.86)	*F* = 19.74	<.001^b^
Gender, No. (%)					
Female	80 (70.18)	73 (70.87)	48 (58.54)	χ^2^ = 15.17	.004
Male	34 (29.82)	30 (29.13)	29 (35.37)		
Nonbinary	NA	NA	5 (6.10)		
Education, No. (%)					
≤High school	17 (14.91)	19 (18.45)	8 (10)	χ^2^ = 25.30	<.001
Some college (≥1 y)	67 (58.78)	53 (51.46)	27 (33)		
Technical school or associate’s degree	0 (0)	2 (1.94)	1 (1)		
≥Bachelor’s degree	30 (26.31)	29 (28.15)	46 (56)		
Race, No. (%)^c^					
Asian	18 (15.69)	21 (20.39)	17 (20.73)	χ^2^ = 9.17	.33
Black or African American	12 (10.53)	11 (10.68)	7 (8.54)		
White	81 (71.05)	64 (62.13)	52 (63.41)		
More than 1 race	3 (2.63)	7 (6.80)	4 (4.88)		
Did not wish to report	0	0	2 (2.44)		
MOODS-SR manic mood domain score, mean (SD)	6.94 (5.55)	7.72 (5.52)	5.06 (4.97)	*F* = 5.75	.004^d^
MOODS-SR depressive mood domain score, mean (SD)	8.43 (7.89)	10.65 (8.67)	6.27 (7.20)	*F* = 6.90	.001^e^
MDD, No. (%)	28 (24.56)	40 (38.84)	11 (13.41)	χ^2^ = 15.50	<.001
Any anxiety disorder, No. (%)	28 (24.56)	44 (42.72)	11 (13.41)	χ^2^ = 20.49	<.001
ADHD, No. (%)	<10^f^	<10^f^	<10^f^	NA	NA
Taking psychotropic medication, No. (%)	<10^f^	<10^f^	0	NA	NA

Abbreviations: ADHD, attention-deficit/hyperactivity disorder; MDD, major depressive disorder; MOODS-SR, Mood Spectrum Self-Report; NA, not applicable.

^a^ Age, gender, education, and lifetime psychiatric diagnoses were assessed at time of functional magnetic resonance imaging scan. Participants may be diagnosed with more than one 1 psychiatric disorder. See the eMethods in Supplement 1 for family history information.

^b^ Test sample 2 was significantly older than the discovery sample and test sample 1; the discovery sample and test sample 1 did not significantly differ on age.

^c^ Race and ethnicity data were collected via multiple-choice questions (including the option to select all that apply), with answer options determined by an National Institutes of Health directive derived from the 2000 US Census results.^49^ These data are included descriptively and were not included in any a priori analyses.

^d^ Test sample 2 had significantly lower MOODS-SR manic mood domain scores than the discovery sample and test sample 1; the discovery sample and test sample 1 did not significantly differ on MOODS-SR manic mood domain scores.

^e^ Test sample 2 had significantly lower MOODS-SR depressive mood domain scores than test sample 1; the discovery sample and test sample 1, and the discovery sample and test sample 2, did not significantly differ on MOODS-SR depressive mood domain scores.

^f^ There were too few participants to provide numbers without compromising identifiability.

Participants’ lifetime mania/hypomania and depression risk were assessed by the MOODS-SR^11^ mood domains. The manic mood domain assessed euphoria, inflated self-esteem, mixed instability or irritability, creativity, sociability or extraversion, and wastefulness or recklessness. The depressive mood domain assessed depressive mood and substance use–related depression.^13^ While other clinical scales assessed present symptom severity as part of a larger study (eMethods in Supplement 1), they were not the focus of this study.

### Functional Magnetic Resonance Imaging Task

Participants completed a facial emotion processing task (eFigure 1 in Supplement 1). The main stimulus contrast was approach-related emotional expressions (angry and happy) vs implicit baseline. For functional magnetic resonance imaging task, acquisition parameters, and preprocessing details, see the eMethods in Supplement 1.

### Activity and Functional Connectivity

Given extant findings highlighting functional alterations in BD predominantly in the DMN, CEN, VAN, and SN prefrontal cortical regions, an anatomically defined region-of-interest mask included the following bilateral regions from these a priori networks: DMN mPFC (Brodmann area [BA] 10), CEN dlPFC (BA9 and BA46), VAN and SN vlPFC (BA47), SN vACC (BA24), and dACC (BA32), as well as OFC (BA11), and other regions connected with the SN and CEN supporting emotional salience, cognition, and motor learning—ie, bilateral amygdala, insula, and striatum (caudate, putamen, and ventral striatum) (spheres at Montreal Neurologic Institute [MNI] coordinates 9, 9, −8 and −9, 9, −8; radius = 8 mm). Parameter estimates of significant clusters of activity within this mask (familywise error *P* < .05; cluster size, *k* > 20 voxels) to our main stimulus contrast were extracted in SPM12 (Statistical Parametric Mapping; The Wellcome Trust Centre for Neuroimaging).

Generalized psychophysiological interaction^50^ calculated significant functional connectivity between anatomically defined bilateral seed regions representing the CEN (dlPFC and caudate), SN (dACC, amygdala, and putamen), and VAN and SN (vlPFC) and targets within the rest of the above region-of-interest mask for the main contrast (familywise error *P* < .05; *k* > 20 voxels). In each sample, we separately identified and extracted parameter estimates of approach-related activity and functional connectivity within the above region-of-interest mask.

### Associations With Mania/Hypomania and Depression Risk

To test hypotheses 1 and 2 in the discovery sample, elastic net–penalized least squares regression for variable selection with 10-fold cross-validation was performed using GLMNET in R version 4.0 (R Foundation) and the appropriate regression family to identify nonzero independent variables in 2 separate risk models: 1 for MOODS-SR mania/hypomania and 1 for MOODS-SR depression (dependent variables). Significant neural measures (activity and functional connectivity) and demographic variables (age and gender) were independent variables. Model coefficients were selected at λ minimum.

Next, in the discovery sample, using SPSS version 27 (IBM) and the 2 separate risk models, the appropriate regression family model assessed the magnitude of the associations between nonzero independent variables and each MOODS-SR DV. Results were corrected for multiple comparisons (false discovery rate [FDR]–adjusted *P* < .05) across both models within each sample.

To further determine each model’s specificity to mania/hypomania or depression risk, all nonzero coefficients from both models were included as independent variables in additional mania/hypomania and depression risk models.

### Replication

To test hypothesis 3, in 2 independent test samples, SPSS regression models using the nonzero independent variables identified from the discovery sample determined whether independent variable–dependent variable associations replicated in the 2 test samples. Standard cross-validation then evaluated the models’ predictive performance in each sample^51^ (eMethods in Supplement 1). Additional risk models were then performed in each test sample as above.

Post hoc sensitivity tests of the above SPSS regression model procedures for the 3 hypotheses were performed in subsets of participants from each sample who were unmedicated and without lifetime diagnoses of major depressive disorder or attention-deficit/hyperactivity disorder, as these disorders can be misdiagnosed as BD^3,52^ and can precede BD and other psychiatric disorders.^52,53,54^ Exploratory analyses assessed neural responses to all facial emotions (angry, happy, sad, and fearful) vs implicit baseline to test whether our main findings were specific to approach-related emotions.

## Results

The patterns of approach-related neural activity and functional connectivity in the 2 test samples were mostly consistent with that shown in the discovery sample (eTable 2 in Supplement 1), which allowed the 2 risk models generated in the discovery sample to be tested for replication in the test samples. Given that the MOODS-SR is a nonnegative count measure and its distributions in each sample were nonnormal (ie, zero inflated and positively skewed) (eTable 1 in Supplement 1), we used Poisson regression models for all analyses.^55,56,57^

### Replicated Associations With Mania/Hypomania Risk

**Figure 1.**
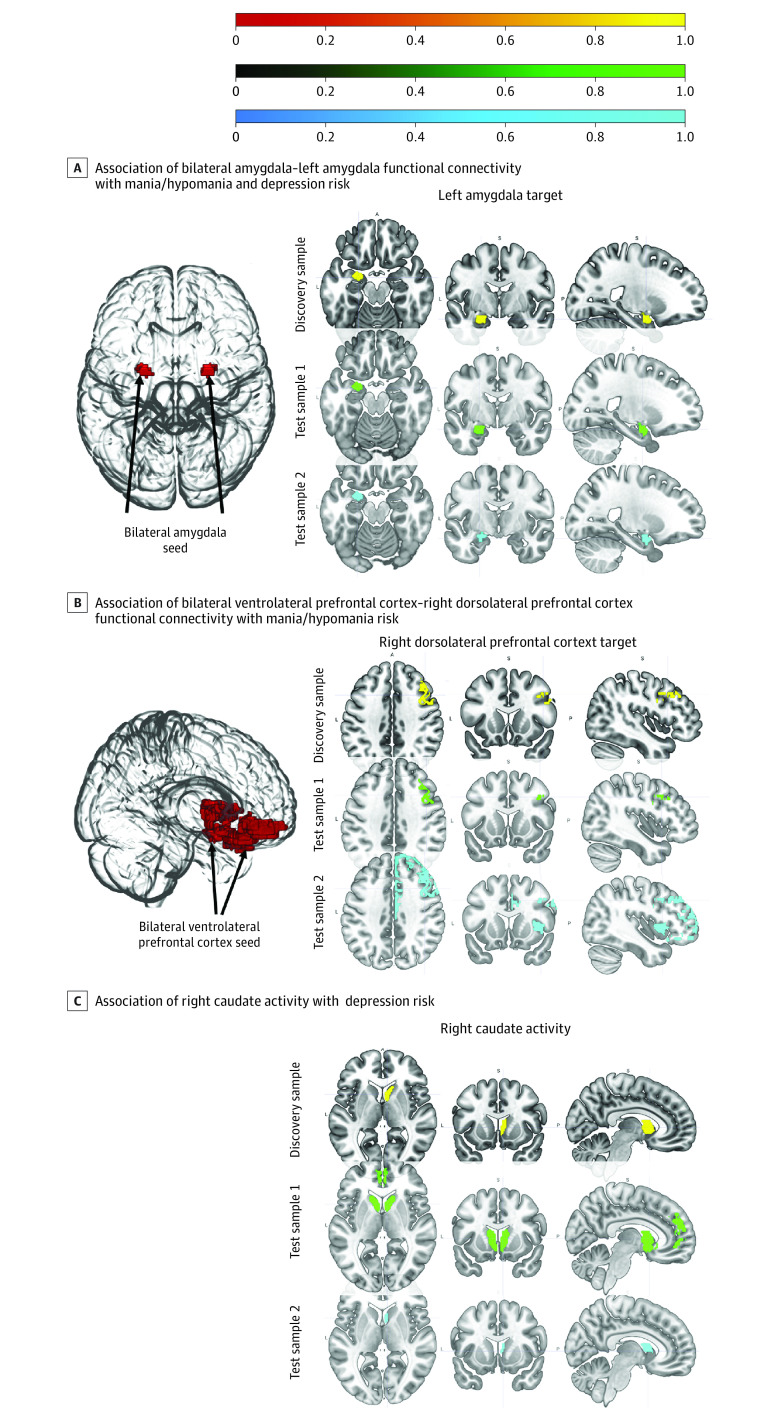
Overview of Replicated Neural Activity and Functional Connectivity in the 3 Samples Neural activity and functional connectivity displayed in the sagittal slices reflect the position in the y-/z-axis. Black arrows highlight the location of the labeled seed regions used in functional connectivity analyses and regions labeled as targets represent the targets identified in the functional connectivity analyses using the specified seeds. A, Arrows point to the bilateral amygdala seed and the target for this analysis was the left amygdala. B, Arrows point to the bilateral ventrolateral prefrontal cortex seed and the target for this analysis was the right dorsolateral prefrontal cortex. Associations were considered significant at familywise error *P* < .05.

**Figure 2.**
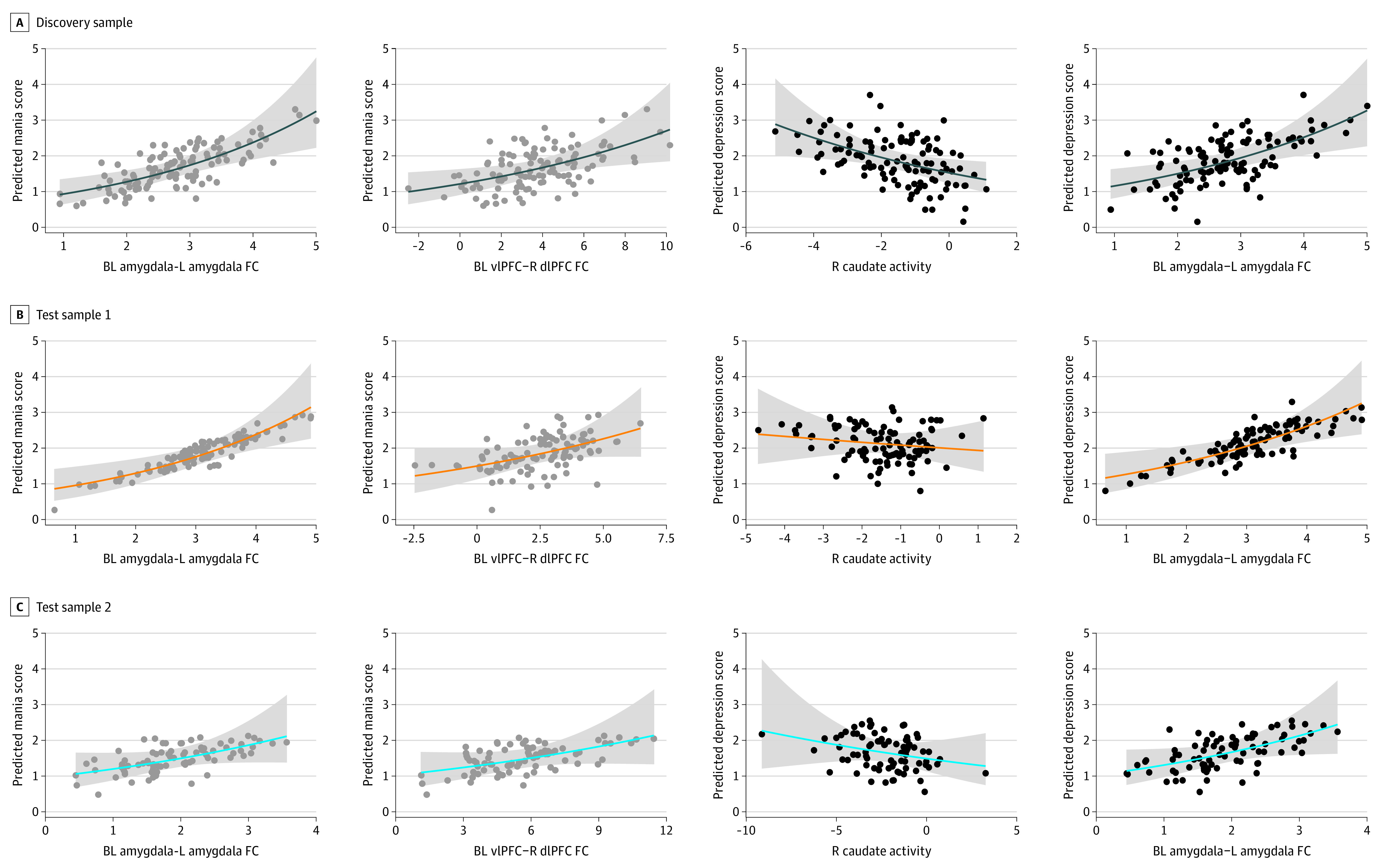
Prediction of Mood Spectrum Self-Report Risk Scores From Replicated Neural Measures in the 3 Samples Scatterplots depict the strength of the contribution from replicated neural measures to predicted Mood Spectrum Self-Report mania or hypomania and depression risk scores. BL indicates bilateral; dlPFC, dorsolateral prefrontal cortex; FC, functional connectivity; L, left; R, right; vlPFC, ventrolateral prefrontal cortex.

In the discovery sample, we first identified neural patterns associated with mania/hypomania risk. Elastic net variable selection after cross-validation using the Poisson family revealed that age, left dlPFC activity, right vlPFC activity, amygdala–left amygdala functional connectivity, vlPFC–right dlPFC functional connectivity, and dACC–left mPFC functional connectivity to approach-related emotions were associated with mania/hypomania risk (Table 2). A Poisson log-link regression model then revealed that left dlPFC (FDR *Q* = 0.003) and right vlPFC (FDR *Q* = 0.007) activity were negatively associated with mania/hypomania risk, and amygdala–left amygdala functional connectivity (Figures 1A and 2A), vlPFC–right dlPFC functional connectivity (Figures 1B and 2A), and dACC–left mPFC functional connectivity (FDR *Q* < 0.001) were positively associated with mania/hypomania risk (Table 2). Age was not significantly associated with mania/hypomania risk.

**Table 2.** Multivariable Regression Model Results With Mania or Hypomania Risk

Parameter	Discovery sample^a^				Test sample 1^b^				Test sample 1^c^			
	β	Hypothesis test		Exponentiated β (95% CI)^d^	β	Hypothesis test		Exponentiated β (95% CI)^d^	β	Hypothesis test		Exponentiated β (95% CI)^d^
		Wald χ^2^	*P* value			Wald χ^2^	*P* value			Wald χ^2^	*P* value	
Age^e^	0.02	0.36	.55	1.02 (0.95-1.10)	0.07	3.75	.05	1.07 (0.10-1.15)	0.03	0.34	.56	1.03 (0.93-1.15)
L dlPFC	−0.06	9.79	.002^f^	0.94 (0.90-0.98)	NA	NA	NA	NA	NA	NA	NA	NA
R vlPFC	−0.14	8.02	.005^f^	0.87 (0.80-0.96)	NA	NA	NA	NA	NA	NA	NA	NA
Amygdala-L amygdala^g^	0.47	236.46	<.001^f^	1.59 (1.50-1.69)	0.50	431.43	<.001^f^	1.65 (1.58-1.73)	0.34	39.37	<.001^f^	1.41 (1.27-1.57)
vlPFC-R dlPFC^g^	0.09	33.62	<.001^f^	1.10 (1.06-1.13)	0.10	16.08	<.001^f^	1.11 (1.05-1.17)	0.07	8.14	.004^f^	1.07 (1.02-1.13)
dACC-L mPFC	0.07	33.53	<.001^f^	1.07 (1.05-1.10)	0.02	0.84	.36	1.02 (0.98-1.06)	0.09	7.02	.008^f^	1.09 (1.02-1.17)

Abbreviations: dACC, dorsal anterior cingulate cortex; dlPFC, dorsolateral prefrontal cortex; L, left; mPFC, medial prefrontal cortex; NA, not applicable; R, right; vlPFC, ventrolateral prefrontal cortex.

^a^ Discovery sample activity and functional connectivity related to MOODS-SR manic mood domain after variable selection with elastic net.

^b^ Test sample 1 activity and functional connectivity related to MOODS-SR manic mood domain.

^c^ Test sample 2 activity and functional connectivity related to MOODS-SR manic mood domain.

^d^ Incidence rate ratio or a 1-unit change in independent variable is an exponentiated β increase in the dependent variable (mania risk score).

^e^ Age was *z* scored for the multivariable regression analyses.

^f^ Raw *P* values were significant at false discovery rate–corrected thresholds; false discovery rate *Q* values are reported in the Results section.

^g^ Finding replicated in all 3 samples.

Two findings replicated in both test samples. Amygdala–left amygdala functional connectivity (Figure 1A) and vlPFC–right dlPFC functional connectivity (Figure 1B) were positively associated with mania/hypomania risk.

In test sample 1, a Poisson log-link regression model revealed that amygdala–left amygdala functional connectivity (Figures 1A and 2B) and vlPFC–right dlPFC functional connectivity (FDR *Q* < 0.001) (Figures 1B and 2B) were positively associated with mania/hypomania risk. Age and dACC–left mPFC functional connectivity were not significantly associated with mania/hypomania risk (Table 2).

In test sample 2, a Poisson log-link regression model revealed that amygdala–left amygdala functional connectivity (FDR *Q* < 0.001) (Figures 1A and 2C), vlPFC–right dlPFC functional connectivity (FDR *Q* = 0.006) (Figures 1B and 2C), and dACC–left mPFC functional connectivity (FDR *Q* = 0.009) were positively associated with mania/hypomania risk. Age was not significantly associated with mania/hypomania risk (Table 2).

Left dlPFC and right vlPFC activity were not observed in either test sample and thus were not included in the above models. Standard cross-validation for mania/hypomania indicated similar predictive performance: discovery sample = 0.75, test sample 1 = 0.74, and test sample 2 = 0.84. For additional mania/hypomania risk models, see the eResults and eTable 3 in Supplement 1.

### Replicated Associations With Depression Risk

In the discovery sample, we tested whether the above neural patterns were common to depression risk. Elastic net variable selection after cross-validation using the Poisson family revealed that age, right vlPFC activity, right caudate activity, amygdala–left amygdala functional connectivity, putamen–right mPFC functional connectivity, putamen–left dACC functional connectivity, dlPFC–right dlPFC functional connectivity, and dACC–left dlPFC functional connectivity to approach-related emotions were associated with depression risk (Table 3). A Poisson log-link regression model then revealed that age (FDR *Q* = 0.01), right vlPFC activity, right caudate activity (Figures 1C and 2A), and dACC–left dlPFC functional connectivity (FDR *Q* < 0.001) were negatively associated with depression risk, and amygdala–left amygdala functional connectivity (Figures 1A and 2A) and dlPFC–right dlPFC functional connectivity (FDR *Q* < 0.001) were positively associated with depression risk (Table 3). Putamen–right mPFC functional connectivity and putamen–left dACC functional connectivity were not significantly associated with depression risk.

**Table 3.** Multivariable Regression Model Results With Depression Risk

Parameter	Discovery sample^a^				Test sample 1^b^				Test sample 2^c^			
	β	Hypothesis test		Exponentiated β (95% CI)^d^	β	Hypothesis test		Exponentiated β (95% CI)^d^	β	Hypothesis test		Exponentiated β (95% CI)^d^
		Wald χ^2^	*P* value			Wald χ^2^	*P* value			Wald χ^2^	*P* value	
Age^e^	−0.09	6.94	.008^f^	0.92 (0.86-0.98)	−0.01	0.06	.80	0.99 (0.93-1.06)	0.24	22.85	<.001^f^	1.27 (1.15-1.40)
R vlPFC	−0.18	26.93	<.001^f^	0.83 (0.78-0.89)	NA	NA	NA	NA	NA	NA	NA	NA
R caudate^g^	−0.16	40.86	<.001^f^	0.86 (0.82-0.90)	−0.19	51.79	<.001^f^	0.83 (0.79-0.87)	−0.10	13.34	<.001^f^	0.91 (0.86-0.96)
Amygdala-L amygdala^g^	0.50	250.08	<.001^f^	1.66 (1.55-1.76)	0.27	61.05	<.001^f^	1.31 (1.23-1.41)	0.39	50.54	<.001^f^	1.48 (1.33-1.66)
dACC-L dlPFC	−0.06	22.85	<.001^f^	0.94 (0.92-0.97)	0.01	0.68	.41	1.01 (0.98-1.05)	0.17	23.36	<.001^f^	1.19 (1.11-1.28)
dlPFC-R dlPFC	0.14	85.15	<.001^f^	1.15 (1.12-1.18)	0.21	54.38	<.001^f^	1.24 (1.17-1.31)	−0.07	1.46	.23	0.93 (0.84-1.04)
putamen-L dACC	−0.02	0.60	.44	0.98 (0.94-1.03)	0.16	51.53	<.001^f^	1.18 (1.13-1.23)	−0.15	10.89	.001^f^	0.86 (0.78-0.94)
putamen-R mPFC	−0.04	2.95	.09	0.96 (0.92-1.01)	NA	NA	NA	NA	0.16	15.32	<.001^f^	1.17 (1.08-1.27)

Abbreviations: dACC, dorsal anterior cingulate cortex; dlPFC, dorsolateral prefrontal cortex; L, left; mPFC, medial prefrontal cortex; NA, not applicable; R, right; vlPFC, ventrolateral prefrontal cortex.

^a^ Discovery sample activity and functional connectivity related to MOODS-SR depressive mood domain after variable selection with elastic net.

^b^ Test sample 1 activity and functional connectivity related to MOODS-SR depressive mood domain.

^c^ Test sample 2 activity and functional connectivity related to MOODS-SR depressive mood domain.

^d^ Incidence rate ratio or a 1-unit change in independent variable is an exponentiated β increase in the dependent variable (depression risk score).

^e^ Age was *z* scored for the multivariable regression analyses.

^f^ Raw *P* values were significant at false discovery rate–corrected thresholds; false discovery rate *Q* values are reported in the Results section.

^g^ Finding replicated in all 3 samples.

Two findings replicated in both test samples. Amygdala–left amygdala functional connectivity (Figure 1A) and right caudate activity (Figure 1C) were positively and negatively associated with depression risk, respectively.

In test sample 1, a Poisson log-link regression model revealed that right caudate activity (Figures 1C and 2B) was negatively associated with depression risk, and amygdala–left amygdala functional connectivity (Figures 1A and 2B), dlPFC–right dlPFC functional connectivity, and putamen–left dACC functional connectivity (FDR *Q* < 0.001) were positively associated with depression risk (Table 3). Age and dACC–left dlPFC functional connectivity were not significantly associated with depression risk. Right vlPFC activity and putamen–right mPFC functional connectivity were not observed in test sample 1 and thus were not included in the model.

In test sample 2, a Poisson log-link regression model revealed that right caudate activity (Figures 1C and 2C) and putamen–left dACC functional connectivity were negatively associated with depression risk, and age, amygdala–left amygdala functional connectivity (Figures 1A and 2C), dACC–left dlPFC functional connectivity, and putamen–right mPFC functional connectivity (FDR *Q* < 0.001) were positively associated with depression risk. dlPFC–right dlPFC functional connectivity was not significantly associated with depression risk (Table 3). Right vlPFC activity was not observed in test sample 2 and thus was not included in the model. Cross-validation for depression revealed similar predictive performance: discovery sample = 0.85, test sample 1 = 1.14, and test sample 2 = 1.08. For additional depression risk models, see eResults and eTable 4 in Supplement 1.

### Post Hoc Analyses

The replicated associations were largely consistent when excluding medicated individuals and individuals with major depressive disorder and attention-deficit/hyperactivity disorder. However, they were not fully replicated in all facial-emotion exploratory analyses, highlighting the specificity of risk markers to approach-related emotions (eResults and eTables 5-13 in Supplement 1).

## Discussion

In this cross-sectional study, we aimed to identify reliable neural markers distinguishing mania/hypomania from depression risk in young adulthood, when psychiatric disorders such as BD often manifest,^2^ via a novel combination of 3 approaches: using an approach-related emotion-processing task, examining large-scale neural networks, and the MOODS-SR to measure mania/hypomania and depression vulnerability. Our findings support our first hypothesis, especially associations between greater amygdala (SN) activity and VAN-CEN functional connectivity and greater mania/hypomania risk; our second hypothesis that distinct neural response patterns would be associated with mania/hypomania vs depression risk; and our third hypothesis that these findings would replicate in 2 independent samples. Specifically, we showed, in 3 independent young adult samples with varying degrees of psychopathology, replicated associations between elevated bilateral amygdala–left amygdala functional connectivity and mania/hypomania and depression risk; greater VAN-CEN (bilateral vlPFC–right dlPFC) functional connectivity and greater mania/hypomania risk; and greater CEN (right caudate) deactivation and greater depression risk.

The amygdala finding parallels animal^58,59,60^ and human imaging^61,62,63^ studies showing synchronous interamygdala activation and functional connectivity and interamygdala coupling during emotion processing in nonclinical populations.^64,65^ In the present study, the left amygdala target extended beyond the amygdala seed mask, possibly extending to the DMN parahippocampal gyrus in the test samples (eFigure 2 in Supplement 1), but the key associations between interamygdala functional connectivity and mania/hypomania and depression risk parallel the above findings. These findings highlight interamygdala functional coupling, possibly reflecting greater attribution of salience to approach-related emotional stimuli, as a potential marker of broader mood disorder risk. Interestingly, bilateral amygdala–left amygdala functional connectivity to all facial emotions was associated with depression risk, but not mania/hypomania risk, in the discovery sample (missing significance in both test samples). Thus, depression risk might be associated with greater attribution of salience to all emotions, with mania/hypomania risk more specifically associated with heightened salience to approach-related emotions, paralleling findings of amygdala hyperactivity to positive emotional stimuli in BD but not unipolar depresson.^25^

By contrast, greater VAN-CEN engagement, indicating greater attention or sensitivity to this specific approach-related emotional context, uniquely characterized mania/hypomania risk, paralleling findings highlighting associations among attentional predispositions toward negative^66^ and positive emotional stimuli,^7,10^ elevated behavioral approach system,^5,6^ and mania/hypomania risk. Our reproducible positive associations between depression risk and caudate deactivation are consistent with reduced CEN function in various contexts shown previously in depression,^32,67,68^ which in turn can be associated with lower emotional regulation capacity.^69,70^ Future studies should further examine these associations and other VAN or CEN regions when testing neural models of BD, as functional alterations in these networks can not only inform studies aiming to replicate our findings in other populations at risk of BD but also help identify prodromal markers in individuals with established BD.

Not all activity or functional connectivity in the discovery sample replicated in both test samples. Bilateral dACC–left mPFC (SN-DMN) functional connectivity was positively associated with mania/hypomania risk in the discovery sample and test sample 2 only, paralleling previous studies in BD showing elevated DMN functional connectivity with other neural networks and thought to result in compromised functioning of these other networks.^71^ Bilateral dlPFC–right dlPFC (CEN-CEN) functional connectivity was positively associated with depression risk in the discovery sample and test sample 1 only, potentially reflecting inefficient, compensatory CEN recruitment and paralleling our previous findings associating elevated CEN activity with depression.^67^ Bilateral dACC–left dlPFC (SN-CEN) functional connectivity and age associations with depression risk in opposite directions in the discovery and test sample 2 and bilateral putamen–dACC (SN-SN) associations with depression risk in opposite directions in the test samples further suggest associations between SN and CEN or motor network functional connectivity, age, and depression risk, although these inconsistent findings should be interpreted with caution, given the directionality differences.

In additional risk models including all nonzero coefficients associated with mania/hypomania and depression risk, caudate deactivation was associated with mania/hypomania and depression risk in all 3 samples whereas vlPFC-dlPFC functional connectivity was associated with depression risk in test sample 1 only, (and these associations were negative, rather than positive, in the other 2 samples), and remained a significant marker of mania/hypomania risk in 2 samples. Caudate deactivation was thus a less specific marker of depression risk than vlPFC-dlPFC functional connectivity was of mania/hypomania risk.

### Limitations

This study had limitations. While a strength was the replication of findings in independent adequately powered samples (eMethods in Supplement 1), future replications are needed. Our findings did not support all (especially CEN-related) network-based hypotheses associated with mania/hypomania risk. Future studies can use tasks that engage the CEN more than our present paradigm. While not all neural measures were identified in all 3 samples, potentially reflecting acquisition parameter differences, we identified robustly replicated neural markers of mania/hypomania and depression risk. While our samples differed on demographic variables and MOODS-SR scores (Table 1), the findings replicated across samples and when using different data analytic pipelines. Sensitivity analyses confirmed that the significant associations generalized to unmedicated individuals and those without major depressive disorder or attention-deficit/hyperactivity disorder. We did not examine neural markers of state anxiety and current mania/hypomania and depression severity, as these were correlated with MOODS-SR measures (eTable 14 and eFigures 3-5 in Supplement 1). We used the MOODS-SR to comprehensively measure future mania/hypomania risk. Future studies can aim to identify neural markers associated with future manic/hypomanic episodes.

## Conclusions

The findings in this study show, in 3 independent samples, robust associations between specific neural markers and mania/hypomania and depression risk. These replicated findings indicate promising neural markers for distinguishing mania/hypomania from depression risk and provide neural targets to guide and monitor interventions in individuals at risk for BD and other affective disorders.

## Notes

Supplement 1. eMethods. Exclusion criteria, power calculation, clinical measures, neuroimaging data acquisition, neuroimaging data preprocessing, functional imaging task, first-level analyses and extraction of neural measures, cross validation procedure eResults. Additional risk models, post-hoc sensitivity analyses, all facial emotions exploratory analyses eFigure 1. Facial emotion-processing task eFigure 2. Comparison of the bilateral amygdala-left amygdala cluster target and bilateral anatomical amygdala seed mask in the three samples eFigure 3. Scatterplots of Pearson correlations between MOODS-SR risk scores and current anxiety, depression and mania/hypomania severity in the Discovery sample eFigure 4. Scatterplots of Pearson correlations between MOODS-SR risk scores and current anxiety, depression and mania/hypomania severity in Test sample I eFigure 5. Scatterplots of Pearson correlations between MOODS-SR risk scores and current anxiety, depression and mania/hypomania severity in Test sample II eFigure 6. Figure 2C, third panel, with N=2 outliers removed eTable 1. MOODS-SR manic and depressive mood domain descriptive statistics eTable 2. Significant activity and functional connectivity to approach-related facial emotions in the three samples eTable 3. Additional mania/hypomania risk models eTable 4. Additional depression risk models eTable 5. Post-hoc sensitivity analyses with mania/hypomania risk in unmedicated participants eTable 6. Post-hoc sensitivity analyses with depression risk in unmedicated participants eTable 7. Post-hoc sensitivity analyses with mania/hypomania risk in participants without MDD eTable 8. Post-hoc sensitivity analyses with depression risk in participants without MDD eTable 9. Post-hoc sensitivity analyses with mania/hypomania risk in participants without ADHD eTable 10. Post-hoc sensitivity analyses with depression risk in participants without ADHD eTable 11. Significant activity and functional connectivity to all facial emotions in the three samples eTable 12. All facial emotions analysis results for mania/hypomania risk eTable 13. All facial emotions analysis results for depression risk eTable 14. MOODS-SR manic and depressive mood domain correlations with state anxiety, depression severity, and mania/hypomania severity eTable 15. Demographic and clinical measures of participants with missing or incomplete data versus participants with complete data from the Discovery sample eTable 16. Demographic and clinical measures of participants with missing or incomplete data versus participants with complete data from Test sample I eTable 17. Demographic and clinical measures of participants with missing or incomplete data versus participants with complete data from Test sample II eTable 18. Family psychiatric history information for the three samples eReferences

Supplement 2. Data sharing statement
