# Supplementary material for: Patterns of Neural Network Functional Connectivity Associated With Mania/Hypomania and Depression Risk in 3 Independent Young Adult Samples
Source: JAMA Psychiatry. 2023 Nov 1;81(2):167–77. doi: 10.1001/jamapsychiatry.2023.4150 (PMC10620679; doi:10.1001/jamapsychiatry.2023.4150)
Supplement: Supplement 1. — eMethods. Exclusion criteria, power calculation, clinical measures, neuroimaging data acquisition, neuroimaging data preprocessing, functional imaging task, first-level analyses and extraction of neural measures, cross validation procedure eResults. Additional risk models, post-hoc sensitivity analyses, all facial emotions exploratory analyses eFigure 1. Facial emotion-processing task eFigure 2. Comparison of the bilateral amygdala-left amygdala cluster target and bilateral anatomical amygdala seed mask in the three samples eFigure 3. Scatterplots of Pearson correlations between MOODS-SR risk scores and current anxiety, depression and mania/hypomania severity in the Discovery sample eFigure 4. Scatterplots of Pearson correlations between MOODS-SR risk scores and current anxiety, depression and mania/hypomania severity in Test sample I eFigure 5. Scatterplots of Pearson correlations between MOODS-SR risk scores and current anxiety, depression and mania/hypomania severity in Test sample II eFigure 6. Figure 2C, third panel, with N=2 outliers removed eTable 1. MOODS-SR manic and depressive mood domain descriptive statistics eTable 2. Significant activity and functional connectivity to approach-related facial emotions in the three samples eTable 3. Additional mania/hypomania risk models eTable 4. Additional depression risk models eTable 5. Post-hoc sensitivity analyses with mania/hypomania risk in unmedicated participants eTable 6. Post-hoc sensitivity analyses with depression risk in unmedicated participants eTable 7. Post-hoc sensitivity analyses with mania/hypomania risk in participants without MDD eTable 8. Post-hoc sensitivity analyses with depression risk in participants without MDD eTable 9. Post-hoc sensitivity analyses with mania/hypomania risk in participants without ADHD eTable 10. Post-hoc sensitivity analyses with depression risk in participants without ADHD eTable 11. Significant activity and functional connectivity to all facial emotions in the three samp [file jamapsychiatry-e234150-s001.pdf]

Supplemental Online Content

Schumer MC, Bertocci MA, Aslam HA, et al. Patterns of neural network functional connectivity associated with mania/hypomania and depression risk in 3 independent young adult samples. *JAMA Psychiatry*. Published online November 1, 2023. doi:10.1001/jamapsychiatry.2023.4150

eMethods. Exclusion criteria, power calculation, clinical measures, neuroimaging data acquisition, neuroimaging data preprocessing, functional imaging task, first-level analyses and extraction of neural measures, cross validation procedure

eResults. Additional risk models, post-hoc sensitivity analyses, all facial emotions exploratory analyses

eFigure 1. Facial emotion-processing task

eFigure 2. Comparison of the bilateral amygdala-left amygdala cluster target and bilateral anatomical amygdala seed mask in the three samples

eFigure 3. Scatterplots of Pearson correlations between MOODS-SR risk scores and current anxiety, depression and mania/hypomania severity in the discovery sample

eFigure 4. Scatterplots of Pearson correlations between MOODS-SR risk scores and current anxiety, depression and mania/hypomania severity in test sample 1

eFigure 5. Scatterplots of Pearson correlations between MOODS-SR risk scores and current anxiety, depression and mania/hypomania severity in test sample 2

eFigure 6. Figure 2C, third panel, with N=2 outliers removed

eTable 1. MOODS-SR manic and depressive mood domain descriptive statistics

eTable 2. Significant activity and functional connectivity to approach-related facial emotions in the three samples

eTable 3. Additional mania/hypomania risk models

eTable 4. Additional depression risk models

eTable 5. Post-hoc sensitivity analyses with mania/hypomania risk in unmedicated participants

© 2023 Schumer MC et al. *JAMA Psychiatry.*

eTable 6. Post-hoc sensitivity analyses with depression risk in unmedicated participants

eTable 7. Post-hoc sensitivity analyses with mania/hypomania risk in participants without MDD

eTable 8. Post-hoc sensitivity analyses with depression risk in participants without MDD

eTable 9. Post-hoc sensitivity analyses with mania/hypomania risk in participants without ADHD

eTable 10. Post-hoc sensitivity analyses with depression risk in participants without ADHD

eTable 11. Significant activity and functional connectivity to all facial emotions in the three samples

eTable 12. All facial emotions analysis results for mania/hypomania risk

eTable 13. All facial emotions analysis results for depression risk

eTable 14. MOODS-SR manic and depressive mood domain correlations with state anxiety, depression severity, and mania/hypomania severity

eTable 15. Demographic and clinical measures of participants with missing or incomplete data versus participants with complete data from the discovery sample

eTable 16. Demographic and clinical measures of participants with missing or incomplete data versus participants with complete data from test sample 1

eTable 17. Demographic and clinical measures of participants with missing or incomplete data versus participants with complete data from test sample 2

eTable 18. Family psychiatric history information for the three samples

eReferences

This supplemental material has been provided by the authors to give readers additional information about their work.

© 2023 Schumer MC et al. *JAMA Psychiatry.*

eMethods. Exclusion criteria, power calculation, clinical measures, neuroimaging data acquisition, neuroimaging data preprocessing, functional imaging task, first-level modeling and extraction of neural measures, cross validation procedure

Exclusion criteria

Discovery sample: *n*=133 participants were scanned on a Siemens Trio scanner; *n*=19 were excluded due to: excessive motion (>4 mm/mean framewise displacement>0.05) (*n*=13), incomplete neuroimaging assessments or missing data (*n*=4), having a diagnosis of Bipolar Disorder (*n*=2). See eTable 15 for comparisons of demographic and clinical measures in included participants versus excluded participants from the discovery sample.

Test sample 1: *n*=136 participants were scanned on a Siemens Prisma scanner; *n*=33 were excluded due to: excessive motion (*n*=17), incomplete neuroimaging assessments or missing data (*n*=13), having a diagnosis of Bipolar Disorder (*n*=3). See eTable 16 for comparisons of demographic and clinical measures in included participants versus excluded participants from test sample 1.

Test sample 2: *n*=106 participants were scanned on a Siemens Prisma scanner; *n*=24 were excluded due to: excessive motion (*n*=9), incomplete neuroimaging assessments or missing data (*n*=15). None of the participants in test sample 2 had a diagnosis of Bipolar Disorder. See eTable 17 for comparisons of demographic and clinical measures in included participants versus excluded participants from test sample 2.

Additional exclusion criteria included: visual disturbance (<20/40 Snellen corrected visual acuity); presence/questionable history of metallic foreign objects in body; positive pregnancy test/self-reporting of pregnancy for female individuals; claustrophobia; Mini-Mental State Examination score<24; a premorbid IQ estimate<85 (determined by the National Adult Reading Test); substance use disorder (SUD) or illicit substance use (except cannabis, given its common usage in young adults) in the prior three months; any history of serious medical/physical conditions: head injury, neurological disorder, pervasive developmental disorder (e.g., autism), history of brain tumor/brain surgery, progressive endocrine disorder, heart disorder, or other major systemic medical conditions (kidney disease, multiple sclerosis, cerebral palsy, blindness, serious physical disability) or chronic/acute condition including any managed by medication (chronic back problem, recent surgery); current psychosis and/or lifetime history of primary psychotic disorder; taking medication for an excluded medical condition; current treatment with psychotropic medication for >2 weeks; previous psychotropic medication treatment in the past three months; inability to comprehend English. All participants were right-handed. The Structured Clinical Interview for DSM-5 Research Version (SCID-5-RV) was administered to all participants to assess for current/past month and lifetime DSM-5 psychiatric disorders[1]. Diagnosis and subsequent exclusion of Bipolar Disorder were defined with DSM-5 criteria, based on administration of the SCID-5-RV. For information on family psychiatric history of the three samples, see eTable 18.

Power calculation

With our sample sizes (discovery sample *n*=114, test sample 1 *n*=103, test sample 2 *n*=82), for continuous outcome measures, we had 90%, 86%, and 75% power, respectively, to detect a significant(P=0.05) effect(f^2=0.15) for the independent variables, controlling for up to five independent variables.

Clinical measures

Mania/hypomania and depression lifetime risk were measured by the Mood Spectrum Self-Report (MOODS-SR)[2–6], a 160-item count measure assessing lifetime occurrence of threshold/subthreshold manic/hypomanic and depressive symptomology, behavioral traits, lifestyles, and features related to mood/affective dysregulation characterizing subsyndromal-prodromal states of affective disturbance. The present study specifically focused on the manic and depressive 'mood' domains, which assess mood lability and associated changes in interest towards interpersonal relationships, work, hobbies, and sports. These scales are included within the MOODS-SR, which also includes five other domain scales: manic energy, depressive energy, manic cognition, depressive cognition, and rhythmicity.

© 2023 Schumer MC et al. *JAMA Psychiatry.*

In addition to the MOODS-SR mania/hypomania and depression risk assessments described in the main text and as above, additional clinical scales measured present symptom severity as part of a larger study (R01MH100041/R37MH100041). These scales included the Young Mania Rating Scale[7] to measure current mania/hypomania severity, the Hamilton Rating Scale for Depression[8] to measure current depression severity, and Hamilton Anxiety Rating Scale[9] to measure state anxiety.

Neuroimaging data acquisition

All neuroimaging data were collected at the University of Pittsburgh.

In the discovery sample, data were collected using a 3.0 Tesla Siemens Trio 2 MRI scanner. Blood-oxygenation-level-dependent (BOLD) images were acquired with a gradient echo echo-planar imaging (EPI) multiband (MB) sequence (18 slices, three-factor multiband (18x(MB)3); 2.3 mm^3 isotropic voxels; TR/TE=1500ms/30 ms; field of view (FOV)=220×220 mm; matrix=96×96; flip angle=55°; bandwidth=1860 Hz Px–1). Structural 3D axial MPRAGE images were acquired in the same session (TR/TE=1500/3.19 ms; FOV=256×256 mm; flip angle=8°; 1 mm^3 isotropic voxels; 176 continuous slices), as were fieldmaps (2.3 mm^3 isotropic voxels; TR=500 ms, TE1=4.92 ms, TE2=7.38 ms; FOV=220×220 mm; flip angle=45°; bandwidth=1302 Hz Px–1).

Data for both independent test samples were collected using a 3.0 Tesla Siemens Prisma MRI scanner. For test sample 1, BOLD images were acquired with a gradient echo EPI multiband (MB)3 sequence covering 18x(MB)3(=54) oblique slices (TR=1500 ms, TE=30 ms; 2.3 mm^3 slices; flip angle 55°; FOV=220x220; matrix=96x96). Structural 3D axial MPRAGE images were acquired with the following parameters (TR=1520 ms, TE=3.17 ms; flip angle 8°; FOV=256x256 mm; 1 mm^3 isotropic voxels; 176 continuous slices). For test sample 2, mean BOLD T2*-weighted images were collected with a multiband multi-echo EPI (MBME)5-factor sequence covering 12x(MBME)5(=70) oblique slices (TR=1500 ms, TE1=15.60 ms, TE2=39.78 ms, TE3=63.96 ms; 2 mm^3 slices; FOV=210x210; matrix=96x96; slice thickness=2 mm; flip angle=70°, bandwidth=2290 Hz/Px), along with structural 3D sagittal MPRAGE images (TR/TE=1520/3.17 ms; FOV=256×256mm; flip angle=8°; 1 mm^3 isotropic voxels; 176 continuous slices) and fieldmaps (TR=731 ms, TE1=4.92 ms, TE2=7.38 ms; flip angle=50°; bandwidth=566 Hz/Px).

Neuroimaging data preprocessing

Imaging data for both the discovery sample and test sample 1 were preprocessed using a combination of software packages (SPM, FSL[10,11], and AFNI[12]) implemented in Nipype[13]. Standard preprocessing steps were applied including realignment to the first volume of the time series, co-registration of realigned images to each participant's anatomic scan, normalization of anatomic images to the MNI152 template, despiking using AFNI 3dDespike, and spatial smoothing using a Full-width at half maximum (FWHM) of 6 mm.

Imaging data for test sample 2 was preprocessed using fMRIPrep[14], FSL (5.0.9), and AFNI 20160207 (RRID:SCR_005927). The first volume of the time series and its skull-stripped version were generated from the shortest echo of the BOLD run using fMRIPrep, followed by co-registration to the T1-weighted reference image using FreeSurfer bbregister and estimation of head-motion parameters using FSL mcflirt. Slice-time correction was performed using AFNI 3dTshift. BOLD time-series were then resampled into native space. Multi-echo (ME) independent components analysis (ME-ICA)[15,16] was then applied to preprocessed BOLD images using Tedana[17] for ME integration. Despiking using AFNI 3dDespike and spatial smoothing using a FWHM of 6 mm were then applied.

Functional imaging task

Participants completed a well-validated facial emotion-processing task (eFigure 1)[18–21]. During the 12.5-minute block design task, participants viewed facial expressions from the NimStim dataset[22] that morphed in 5% increments from neutral (0% emotion) to angry, fear, sad, or happy (100% emotion) over the course of a one-second movie. In control trials, movies comprised a simple dark oval superimposed on a light-grey oval that subsequently morphs into a larger shape. There were three blocks for each of the four face emotions (happy, fear, angry, and sad) with 12 stimuli per block, and six control blocks with six stimuli per block. Blocks were presented in a pseudorandomized

© 2023 Schumer MC et al. *JAMA Psychiatry.*

order. Participants used one of three fingers to press a button indicating the color of a semi-transparent foreground color flash (orange, blue, or yellow) that appeared during the mid 200-650 ms of the one-second face movie.

First-level modeling and extraction of neural measures

SPM8 (discovery sample and test sample 1) and SPM12 (test sample 2) were used to generate a first-level fixed-effects general linear model for each participant in each sample. In all samples, the model included six motion parameters and a regressor controlling for physiologic noise derived from the mean signal of white matter and CSF as covariates of no interest. A high-pass frequency filter (256 seconds) and autoregressive modeling (AR(1) for discovery sample and test sample 1, FAST for test sample 2) were also applied. In all samples, from the first-level activation maps, statistical parametric maps were generated for our emotion contrast of interest, the main effect of approach-related emotions (angry and happy faces combined) versus implicit baseline, to identify neural activity and functional connectivity related to processing approach-related emotions across the entire sample using a one sample *t*-test in SPM12. In all samples, parameter estimates for clusters in which there was significant activity were then extracted using a family-wise error (FWE) correction threshold of α=0.05, within an anatomical region-of-interest (ROI) mask that was created in SPM using the WFU Pickatlas, Wake Forest University, Winston-Salem[23] (further described in the main text Methods 'Activity and Functional Connectivity' section).

Cross-validation procedure

Standard cross-validation evaluated the replication of the risk models' predictive ability[24] in all three samples via comparing the average in-sample and out-of-sample difference between predicted MOODS-SR scores and actual MOODS-SR scores. Raw MOODS-SR scores were natural log-transformed and scaled up by 1 to account for scores of 0. Formula: mean(abs(ln(raw MOODS-SR score+1)-predicted MOODS-SR score)).

© 2023 Schumer MC et al. *JAMA Psychiatry.*

eResults. Additional risk models, post-hoc sensitivity analyses, all facial emotions exploratory analysis

Additional mania/hypomania risk models

In the discovery sample, a Poisson log-link regression model revealed that left dorsolateral prefrontal cortex(dlPFC) and right caudate activity were negatively, and amygdala-left amygdala functional connectivity, dorsal anterior cingulate cortex(dACC)-left medial prefrontal cortex(mPFC) functional connectivity, and dlPFC-right dlPFC functional connectivity were positively, associated with mania/hypomania risk (FDR q<0.001)(eTable 3A). Age, right ventrolateral prefrontal cortex(vlPFC) activity, vlPFC-right dlPFC functional connectivity, dACC-left dlPFC functional connectivity, putamen-right mPFC functional connectivity, and putamen-left dACC functional connectivity were not significantly associated with mania/hypomania risk.

In test sample 1, a Poisson log-link regression model revealed that right caudate activity (FDR q<0.001) was negatively, and amygdala-left amygdala functional connectivity (FDR q<0.001) and vlPFC-right dlPFC functional connectivity (FDR q=0.004) were positively, associated with mania/hypomania risk (eTable 3B). Age, dACC-left mPFC functional connectivity, dACC-left dlPFC functional connectivity, dlPFC-right dlPFC functional connectivity, and putamen-left dACC functional connectivity were not significantly associated with mania/hypomania risk. Left dlPFC activity, right vlPFC activity, and putamen-right mPFC functional connectivity were not observed in test sample 1 and were thus not included in the model.

In test sample 2, a Poisson log-link regression model revealed that right caudate activity (FDR q=0.002) was negatively, and amygdala-left amygdala functional connectivity (FDR q<0.001) and dlPFC-right dlPFC functional connectivity (FDR q=0.021) were positively, associated with mania/hypomania risk (eTable 3C). Age, vlPFC-right dlPFC functional connectivity, dACC-left mPFC/dlPFC functional connectivity, putamen-left dACC functional connectivity, and putamen-right mPFC functional connectivity were not significantly associated with mania/hypomania risk. Left dlPFC activity and right vlPFC activity were not observed in test sample 2 and were thus not included in the model.

Additional depression risk models

In the discovery sample, a Poisson log-link regression model revealed that left dlPFC activity, right caudate activity, and dACC-left dlPFC functional connectivity (FDR q<0.001) were negatively, and amygdala-left amygdala functional connectivity (FDR q<0.001), dACC-left mPFC functional connectivity (FDR q=0.002), and dlPFC-right dlPFC functional connectivity (FDR q<0.001) were positively, associated with depression risk (eTable 4A). Age, right vlPFC activity, vlPFC-right dlPFC functional connectivity, putamen-right mPFC functional connectivity, and putamen-left dACC functional connectivity were not significantly associated with depression risk.

In test sample 1, a Poisson log-link regression model revealed that right caudate activity (FDR q<0.001) was negatively, and amygdala-left amygdala functional connectivity (FDR q<0.001), vlPFC-right dlPFC functional connectivity (FDR q=0.002), dlPFC-right dlPFC functional connectivity (FDR q<0.001), and putamen-left dACC functional connectivity (FDR q<0.001) were positively, associated with depression risk (eTable 4B). Age, dACC-left mPFC functional connectivity, and dACC-left dlPFC functional connectivity were not significantly associated with depression risk. Left dlPFC activity, right vlPFC activity, and putamen-right mPFC functional connectivity were not observed in test sample 1 and were thus not included in the model.

In test sample 2, a Poisson log-link regression model revealed that age, amygdala-left amygdala functional connectivity, and dACC-left mPFC/dlPFC functional connectivity (FDR q<0.001) were positively, and right caudate activity, putamen-right mPFC functional connectivity (FDR q<0.001), and putamen-left dACC functional connectivity (FDR q=0.002) were negatively, associated with depression risk (eTable 4C). vlPFC-right dlPFC functional connectivity and dlPFC-right dlPFC functional connectivity were not significantly associated with depression risk. Left dlPFC activity and right vlPFC activity were not observed in test sample 2 and were thus not included in the model.

Post-hoc sensitivity analysis: excluding participants taking medication

© 2023 Schumer MC et al. *JAMA Psychiatry.*

In the discovery sample, after excluding participants taking medication, a Poisson log-link regression model revealed that amygdala-left amygdala functional connectivity, vlPFC-right dlPFC functional connectivity, and dACC-left mPFC functional connectivity were positively associated with mania/hypomania risk (FDR $q<0.001$); left dlPFC activity (FDR $q=0.003$) and right vlPFC activity (FDR $q=0.008$) were negatively associated with mania/hypomania risk (eTable 5A). Age was not significantly associated with mania/hypomania risk.

In test sample 1, after excluding participants taking medication, a Poisson log-link regression model revealed that amygdala-left amygdala functional connectivity and vlPFC-right dlPFC functional connectivity were positively associated with mania/hypomania risk (FDR $q<0.001$) (eTable 5B). Age and dACC-left mPFC functional connectivity were not significantly associated with mania/hypomania risk. Left dlPFC activity and right vlPFC activity were not observed in test sample 1 and were thus not included in the model.

In the discovery sample, after excluding participants taking medication, a Poisson log-link regression model revealed that right vlPFC activity (FDR $q=0.02$), right caudate activity (FDR $q<0.001$), and dACC-left dlPFC functional connectivity (FDR $q<0.001$) were negatively associateed with depression risk; dlPFC-right dlPFC functional connectivity and amygdala-left amygdala functional connectivity were positively associated with depression risk (FDR $q<0.001$) (eTable 6A). Age, putamen-left dACC functional connectivity, and putamen-right mPFC functional connectivity were not significantly associated with depression risk.

In test sample 1, after excluding participants taking medication, a Poisson log-link regression model revealed that right caudate activity was negatively, and amygdala-left amygdala functional connectivity, dlPFC-right dlPFC functional connectivity, and putamen-left dACC functional connectivity were positively, associated with depression risk (FDR $q<0.001$) (eTable 6B). Age and dACC-left dlPFC functional connectivity were not significantly associated with depression risk. Right vlPFC activity and putamen-right mPFC functional connectivity were not observed in test sample 1 and were thus not included in the model.

None of the participants in test sample 2 were taking psychotropic medication.

Post-hoc sensitivity analysis: excluding participants with Major Depressive Disorder (MDD)

In the discovery sample, after excluding participants with MDD, a Poisson log-link regression model revealed that age (FDR $q=0.036$), amygdala-left amygdala functional connectivity, vlPFC-right dlPFC functional connectivity, and dACC-left mPFC functional connectivity were positively associated with mania/hypomania risk (FDR $q<0.001$); left dlPFC activity (FDR $q=0.038$) was negatively associated with mania/hypomania risk (eTable 7A). Right vlPFC activity was not significantly associated with mania/hypomania risk.

In test sample 1, after excluding participants with MDD, a Poisson log-link regression model revealed that amygdala-left amygdala functional connectivity and vlPFC-right dlPFC functional connectivity (FDR $q<0.001$) were positively associated with mania/hypomania risk; age was marginally positively associated with mania/hypomania risk (FDR $q=0.05$) (eTable 7B). dACC-left mPFC functional connectivity was not significantly associated with mania/hypomania risk. Left dlPFC activity and right vlPFC activity were not observed in test sample 1 and were thus not included in the model.

In test sample 2, after excluding participants with MDD, a Poisson log-link regression model revealed that amygdala-left amygdala functional connectivity and vlPFC-right dlPFC functional connectivity were positively associated with mania/hypomania risk (FDR $q<0.001$); age and dACC-left dlPFC functional connectivity were not significantly associated with mania/hypomania risk (eTable 7C). Left dlPFC activity and right vlPFC activity were not observed in test sample 2 and were thus not included in the model.

In the discovery sample, after excluding participants with MDD, a Poisson log-link regression model revealed that age (FDR $q=0.028$), right caudate activity, dACC-left dlPFC functional connectivity, and putamen-right mPFC functional connectivity (FDR $q<0.001$) were negatively associated with depression risk; amygdala-left amygdala functional connectivity, dlPFC-right dlPFC functional connectivity (FDR $q<0.001$), and putamen-left dACC functional connectivity (FDR $q=0.008$) were positively associated with depression risk (eTable 8A). Right vlPFC activity was not significantly associated with depression risk.

© 2023 Schumer MC et al. *JAMA Psychiatry.*

In test sample 1, after excluding participants with MDD, a Poisson log-link regression model revealed that right caudate activity (FDR $q<0.001$) and amygdala-left amygdala functional connectivity (FDR $q=0.013$) were negatively associated with depression risk; dlPFC-right dlPFC functional connectivity and putamen-left dACC functional connectivity (FDR $q<0.001$) were positively associated with depression risk (eTable 8B). Age and dACC-left dlPFC functional connectivity were not significantly associated with depression risk. Right vlPFC activity and putamen-right mPFC functional connectivity were not observed in test sample 1 and were thus not included in the model.

In test sample 2, after excluding participants with MDD, a Poisson log-link regression model revealed that right caudate activity was negatively associated with depression risk (FDR $q<0.001$) and age, amygdala-left amygdala functional connectivity (FDR $q<0.001$), and dACC-left dlPFC functional connectivity (FDR $q=0.002$) were positively associated with depression risk (eTable 8C). dlPFC-right dlPFC functional connectivity, putamen-left dACC functional connectivity, and putamen-right mPFC functional connectivity were not significantly associated with depression risk. Right vlPFC activity was not observed in test sample 2 and was thus not included in the model.

Post-hoc sensitivity analysis: excluding participants with Attention Deficit Hyperactivity Disorder (ADHD)

In the discovery sample, after excluding participants with ADHD, a Poisson log-link regression model revealed that left dlPFC activity (FDR $q=0.024$) and right vlPFC activity (FDR $q=0.009$) were negatively associated with mania/hypomania risk; amygdala-left amygdala functional connectivity, vlPFC-right dlPFC functional connectivity, and dACC-left mPFC functional connectivity (FDR $q<0.001$) were positively associated with mania/hypomania risk (eTable 9A). Age was not significantly associated with mania/hypomania risk.

In test sample 1, after excluding participants with ADHD, a Poisson log-link regression model revealed that amygdala-left amygdala functional connectivity and vlPFC-right dlPFC functional connectivity were positively associated with mania/hypomania risk (FDR $q<0.001$) (eTable 9B). dACC-left mPFC functional connectivity was not significantly associated with mania/hypomania risk, and age was no longer significantly positively associated with mania/hypomania risk after FDR correction ($P=0.048$; FDR $q=0.068$). Left dlPFC activity and right vlPFC activity were not observed in test sample 1 and were thus not included in the model.

In test sample 2, after excluding participants with ADHD, a Poisson log-link regression model revealed that amygdala-left amygdala functional connectivity (FDR $q<0.001$), vlPFC-right dlPFC functional connectivity (FDR $q=0.005$), and dACC-left mPFC functional connectivity (FDR $q=0.017$) were positively associated with mania/hypomania risk (eTable 9C). Age was not significantly associated with mania/hypomania risk. Left dlPFC activity and right vlPFC activity were not observed in test sample 2 and were thus not included in the model.

In the discovery sample, after excluding participants with ADHD, a Poisson log-link regression model revealed that right vlPFC activity (FDR $q=0.007$), right caudate activity and dACC-left dlPFC functional connectivity (FDR $q<0.001$) were negatively associated with depression risk; amygdala-left amygdala functional connectivity and dlPFC-right dlPFC functional connectivity (FDR $q<0.001$) were positively associated with depression risk (eTable 10A). Age, putamen-left dACC functional connectivity, and putamen-right mPFC functional connectivity were not significantly associated with depression risk.

In test sample 1, after excluding participants with ADHD, a Poisson log-link regression model revealed that right caudate was negatively associated with depression risk (FDR $q<0.001$) and amygdala-left amygdala functional connectivity, dlPFC-right dlPFC functional connectivity, and putamen-left dACC functional connectivity (FDR $q<0.001$) were positively associated with depression risk (eTable 10B). Age and dACC-left dlPFC functional connectivity were not significantly associated with depression risk. Right vlPFC activity and putamen-right mPFC functional connectivity were not observed in test sample 1 and were thus not included in the model.

In test sample 2, after excluding participants with ADHD, a Poisson log-link regression model revealed that age, amygdala-left amygdala functional connectivity, dACC-left dlPFC functional connectivity, and putamen-right mPFC functional connectivity were positively associated with depression risk (FDR $q<0.001$); right caudate activity (FDR $q<0.001$) and putamen-left dACC functional connectivity (FDR $q=0.002$) were negatively associated with depression risk (eTable 10C). dlPFC-right dlPFC functional connectivity was not significantly associated with depression risk. Right vlPFC activity was not observed in test sample 2 and was thus not included in the model.

© 2023 Schumer MC et al. *JAMA Psychiatry.*

All facial emotions exploratory analysis

The patterns of neural activity and functional connectivity to the all facial emotions contrast in the two test samples were mostly consistent with that shown in the discovery sample (eTable 11), which allowed the two MOODS-SR risk models generated in the discovery sample to be tested for replication in the two independent test samples using the same procedures described in the main text for the main contrast i.e., approach-related emotions.

First, in the discovery sample, elastic-net variable selection after cross-validation using the Poisson family identified age, right vlPFC activity, right dlPFC activity, caudate-left caudate functional connectivity, dACC-right vlPFC functional connectivity, dlPFC-right insula functional connectivity, and vlPFC-right dlPFC functional connectivity to the all facial emotions contrast as measures associated with mania/hypomania risk (eTable 12A). A Poisson log-link regression model then revealed that age, dACC-right vlPFC functional connectivity, and dlPFC-right insula functional connectivity (FDR $q<0.001$) were positively associated with mania/hypomania risk; right vlPFC activity (FDR $q<0.001$) and right dlPFC activity (FDR $q=0.001$) were negatively associated with mania/hypomania risk (eTable 12A). Caudate-left caudate functional connectivity and vlPFC-right dlPFC functional connectivity were not significantly associated with mania/hypomania risk.

In test sample 1, a Poisson log-link regression model revealed that age(FDR $q<0.001$), dACC-right vlPFC functional connectivity (FDR $q=0.013$), and vlPFC-right dlPFC functional connectivity (FDR $q=0.004$) were positively associated with mania/hypomania risk(eTable 12B). Right dlPFC activity, caudate-left caudate functional connectivity, and dlPFC-right insula functional connectivity were not significantly associated with mania/hypomania risk. Right vlPFC activity was not observed in test sample 1 and was thus not included in the model.

In test sample 2, a Poisson log-link regression model revealed that age and caudate-left caudate functional connectivity (FDR $q<0.001$) were positively associated with mania/hypomania risk (eTable 12C). dACC-right vlPFC functional connectivity and vlPFC-right dlPFC functional connectivity were not significantly associated with mania/hypomania risk. Right vlPFC activity, right dlPFC activity, and dlPFC-right insula functional connectivity were not observed in test sample 2 and were thus not included in the model.

Next, in the discovery sample, elastic-net variable selection after cross-validation using the Poisson family identified gender, age, right vlPFC activity, right caudate activity, amygdala-left amygdala functional connectivity, caudate-left caudate functional connectivity, dACC-left dlPFC functional connectivity, dlPFC-right dlPFC functional connectivity, putamen-right OFC functional connectivity, and putamen-left dACC functional connectivity to the all facial emotions contrast as measures associated with depression risk (eTable 13A). A Poisson log-link regression model then revealed that gender, right vlPFC activity, right caudate activity (FDR $q<0.001$) and dACC-left dlPFC functional connectivity (FDR $q=0.024$) were negatively associated with depression risk; age, amygdala-left amygdala functional connectivity, and dlPFC-right dlPFC functional connectivity (FDR $q<0.001$) were positively associated with depression risk (eTable 13A). Caudate-left caudate functional connectivity, putamen-right OFC functional connectivity, and putamen-left dACC functional connectivity were not significantly associated with depression risk.

In test sample 1, a Poisson log-link regression model revealed that gender (FDR $q=0.006$) and right caudate activity (FDR $q=0.033$) were negatively associated with depression risk; age (FDR $q<0.001$) and caudate-left caudate functional connectivity (FDR $q=0.026$) were positively associated with depression risk (eTable 13B). Amygdala-left amygdala functional connectivity, dACC-left dlPFC functional connectivity, and dlPFC-right dlPFC functional connectivity were not significantly associated with depression risk. Right vlPFC activity, putamen-right OFC functional connectivity, and putamen-left dACC functional connectivity were not observed in test sample 1 and were thus not included in the model.

In test sample 2, a Poisson log-link regression model revealed that age (FDR $q<0.001$), dACC-left dlPFC functional connectivity (FDR $q=0.011$), and putamen-right OFC functional connectivity (FDR $q<0.001$) were positively associated with depression risk; dlPFC-right dlPFC functional connectivity and putamen-left dACC functional connectivity (FDR $q<0.001$) were negatively associated with depression risk (eTable 13C). Gender, right caudate activity, amygdala-left amygdala functional connectivity, and caudate-left caudate functional connectivity were not

© 2023 Schumer MC et al. *JAMA Psychiatry.*

significantly associated with depression risk. Right vlPFC activity was not observed in test sample 2 and was thus not included in the model.

© 2023 Schumer MC et al. *JAMA Psychiatry.*

eFigure 1. Facial emotion-processing task. The top panel represents a happy trial of our facial emotion-processing task. Over a one-second duration, the face changed in 5% increments from neutral (0% emotion) to a happy, sad, angry, or fearful face (100% emotion). The bottom panel represents the changing shape condition. In both cases, participants were asked to identify the color flash presented in the middle of the dynamic change.

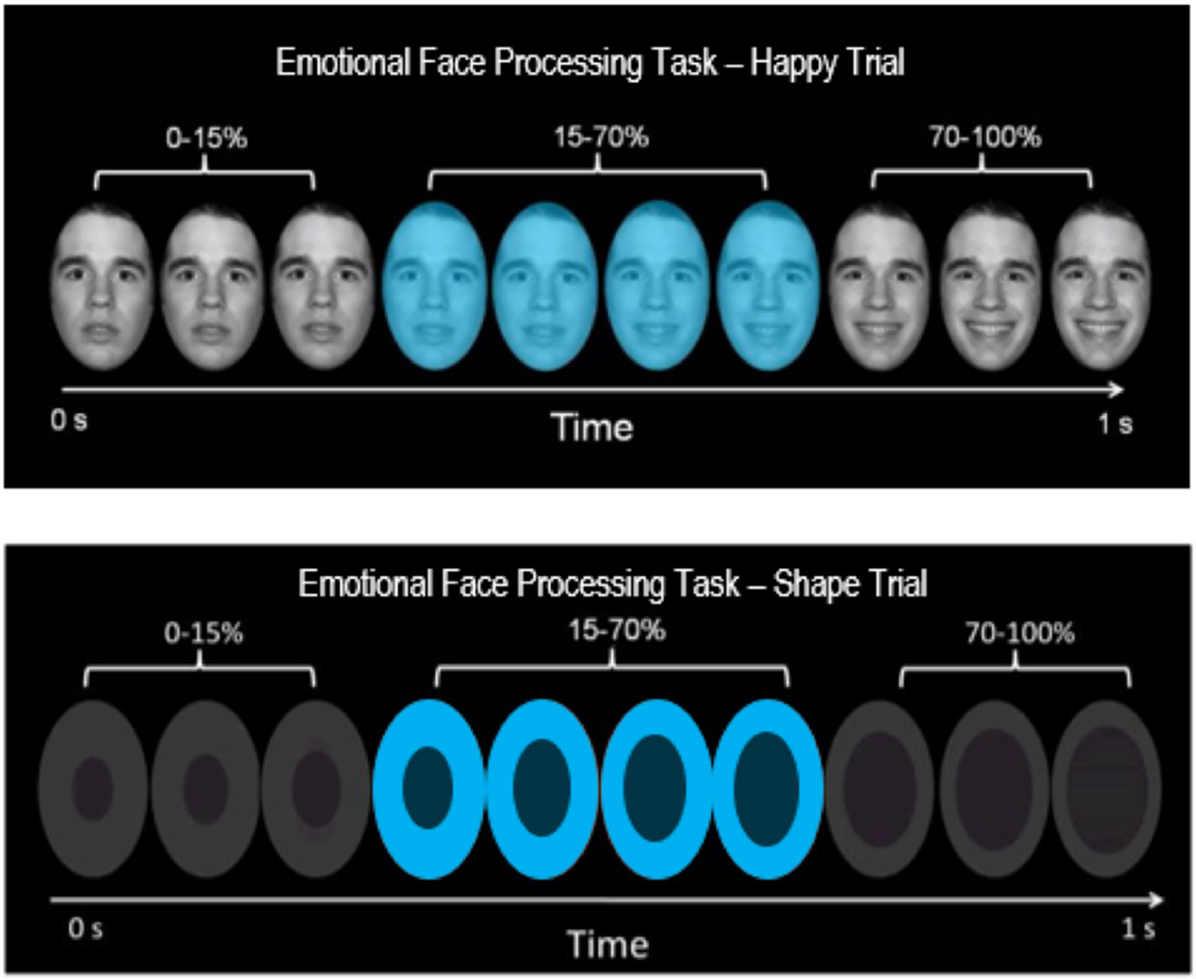

© 2023 Schumer MC et al. *JAMA Psychiatry.*

eFigure 2. Comparison of the bilateral amygdala-left amygdala cluster target and bilateral anatomical amygdala seed mask in the three samples. Black arrows highlight the location of the bilateral amygdala seed mask used in the functional connectivity analysis (shown on the left in red) which is overlaid on each sample's left amygdala cluster target from the bilateral amygdala-left amygdala functional connectivity finding that was associated with both mania/hypomania and depression risk in all three samples. Neural activity and functional connectivity displayed in the sagittal slices reflect the position in the y/z axis.

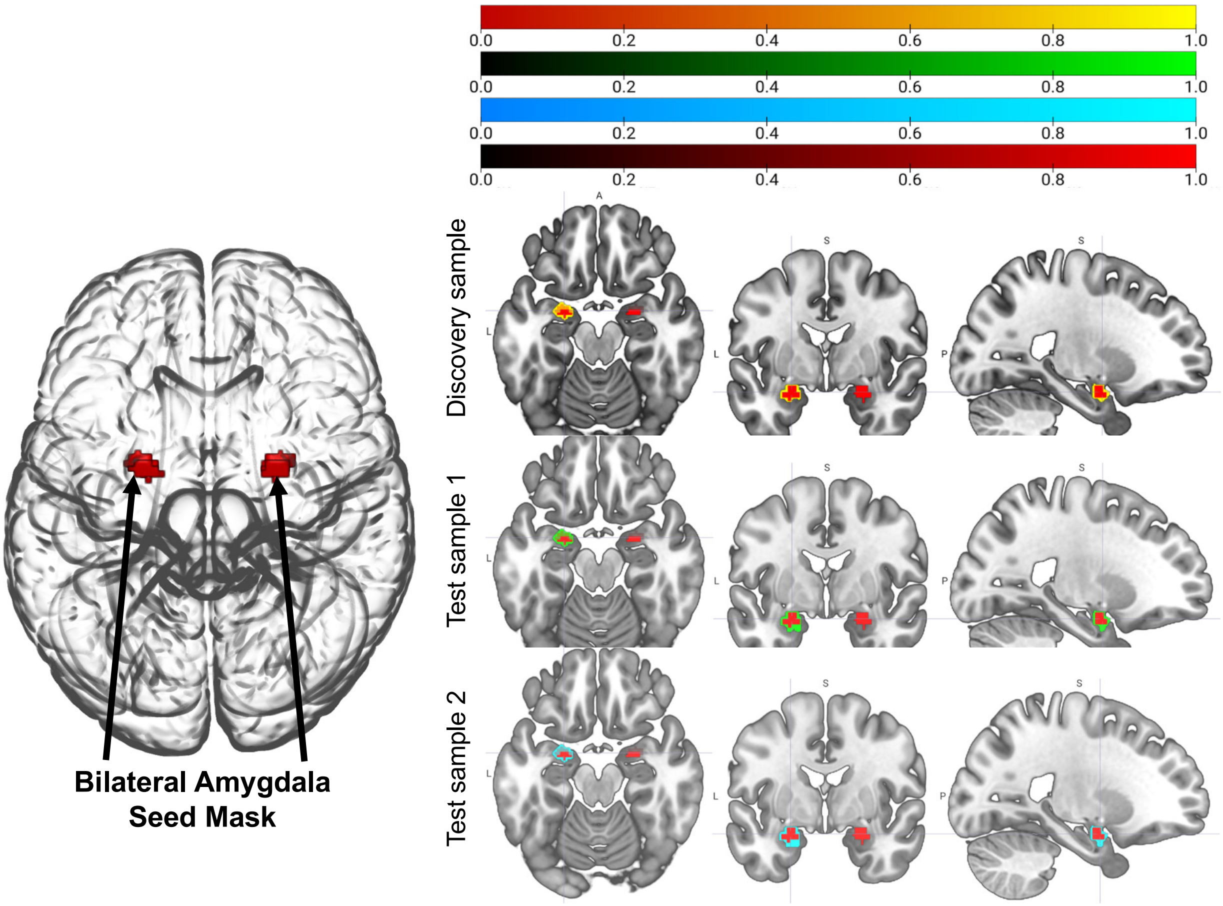

© 2023 Schumer MC et al. *JAMA Psychiatry.*

eFigure 3. Scatterplots of Pearson correlations between MOODS-SR risk scores and current anxiety, depression and mania/hypomania severity in the discovery sample. Correlations with the MOODS-SR manic mood domain are on the left; correlations with the MOODS-SR depressive mood domain are on the right. Abbreviations: HAMA=Hamilton Anxiety Rating Scale; HRSD=Hamilton Rating Scale for Depression; MOODS-SR=Mood Spectrum Self-Report; YMRS=Young Mania Rating Scale.

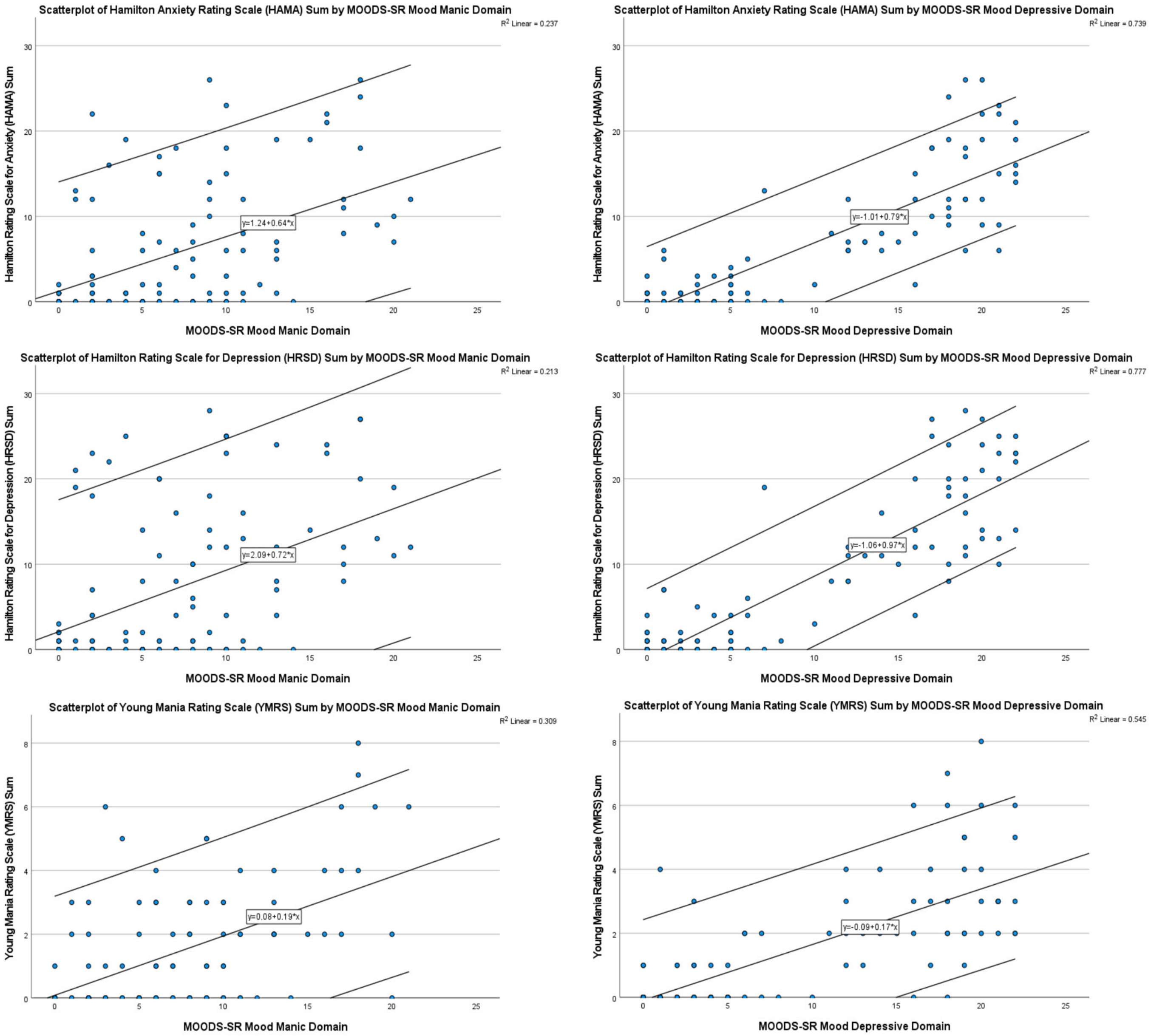

© 2023 Schumer MC et al. *JAMA Psychiatry.*

eFigure 4. Scatterplots of Pearson correlations between MOODS-SR risk scores and current anxiety, depression and mania/hypomania severity in test sample 1. Correlations with the MOODS-SR manic mood domain are on the left; correlations with the MOODS-SR depressive mood domain are on the right. Abbreviations: HAMA=Hamilton Anxiety Rating Scale; HRSD=Hamilton Rating Scale for Depression; MOODS-SR=Mood Spectrum Self-Report; YMRS=Young Mania Rating Scale.

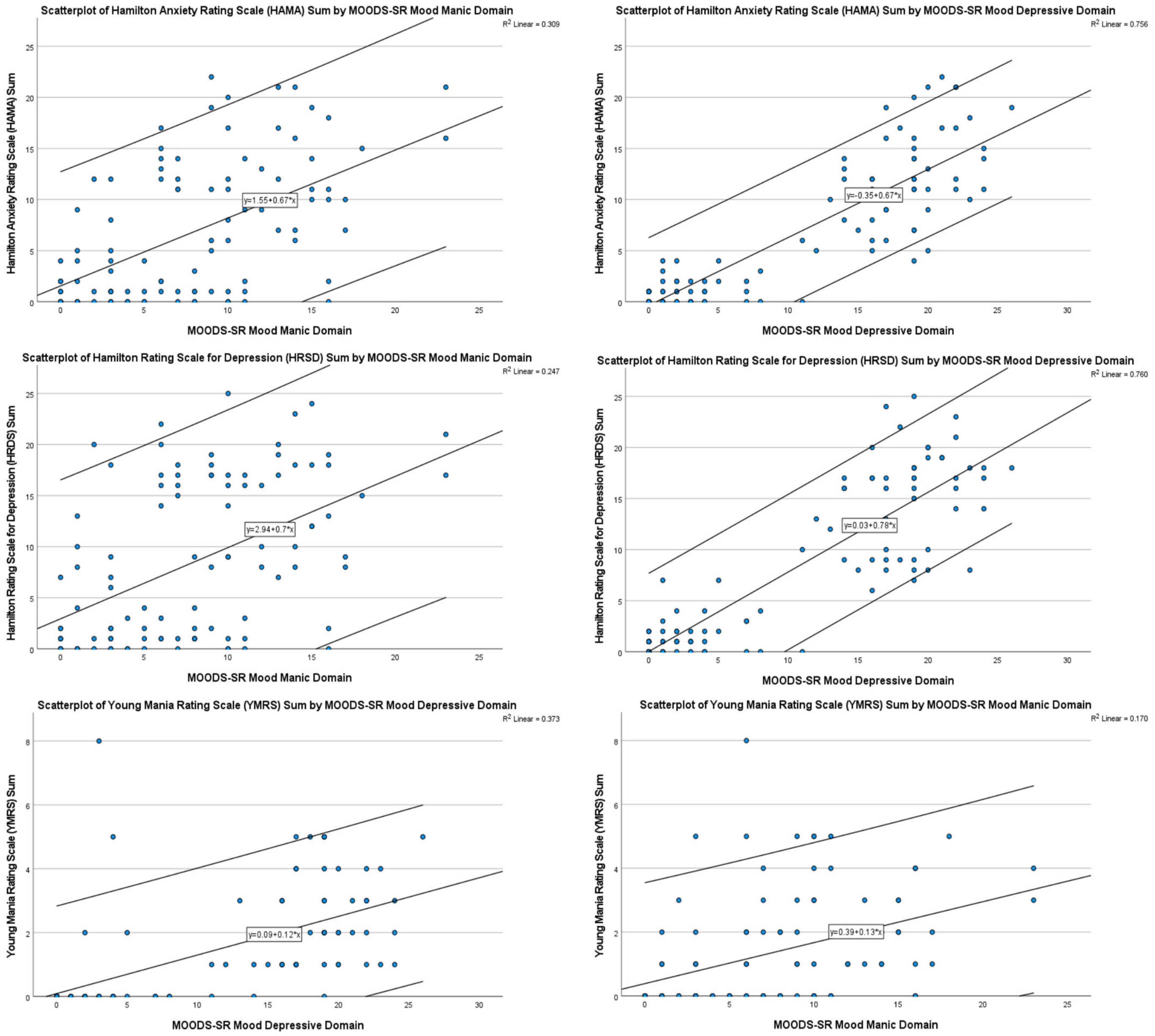

© 2023 Schumer MC et al. *JAMA Psychiatry.*

eFigure 5. Scatterplots of Pearson correlations between MOODS-SR risk scores and current anxiety, depression and mania/hypomania severity in test sample 2. Correlations with the MOODS-SR manic mood domain are on the left; correlations with the MOODS-SR depressive mood domain are on the right. Abbreviations: HAMA=Hamilton Anxiety Rating Scale; HRSD=Hamilton Rating Scale for Depression; MOODS-SR=Mood Spectrum Self-Report; YMRS=Young Mania Rating Scale.

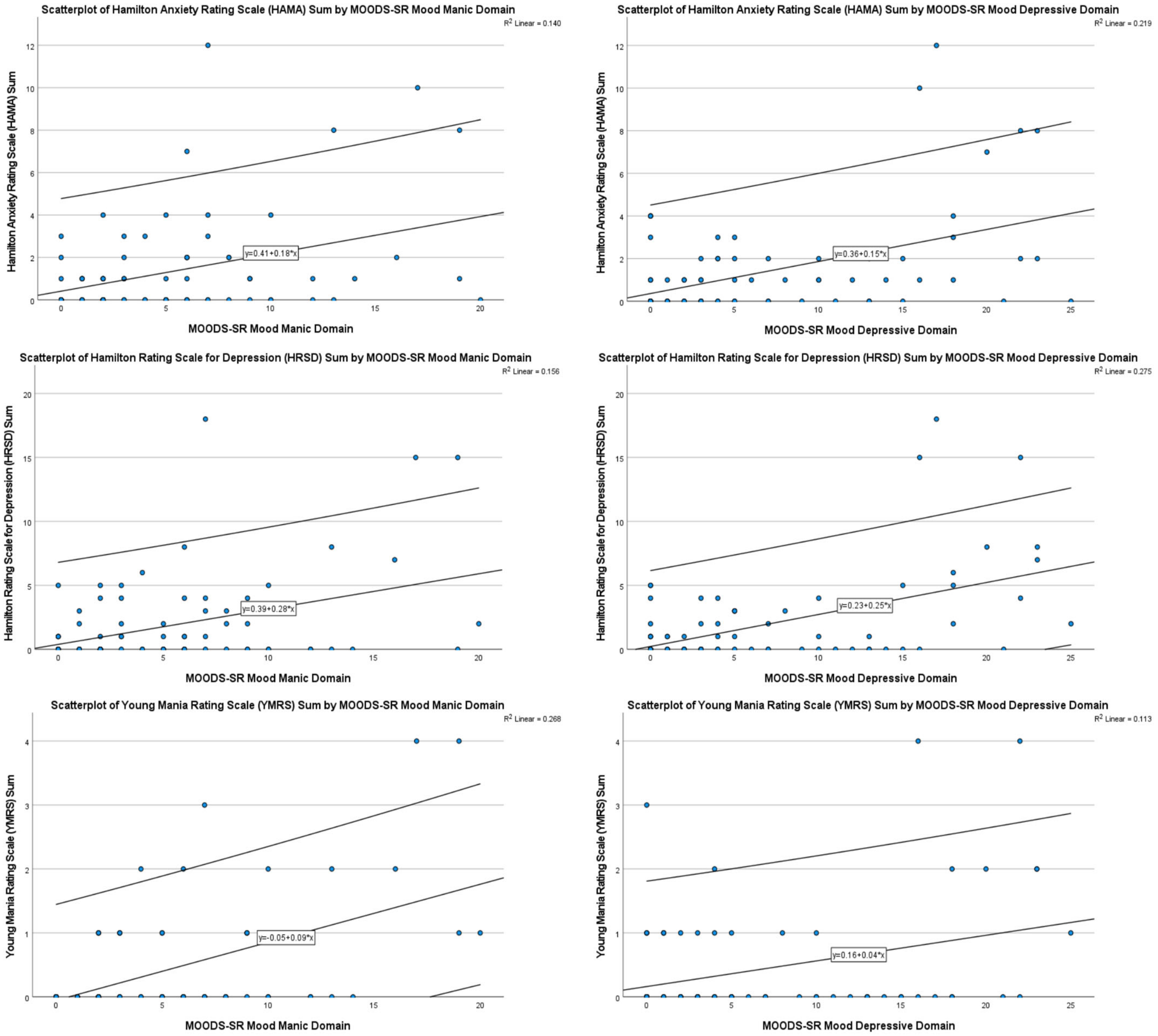

© 2023 Schumer MC et al. *JAMA Psychiatry.*

eFigure 6. Figure 2C, third panel, with *N*=2 outliers removed. Abbreviations: R=Right.

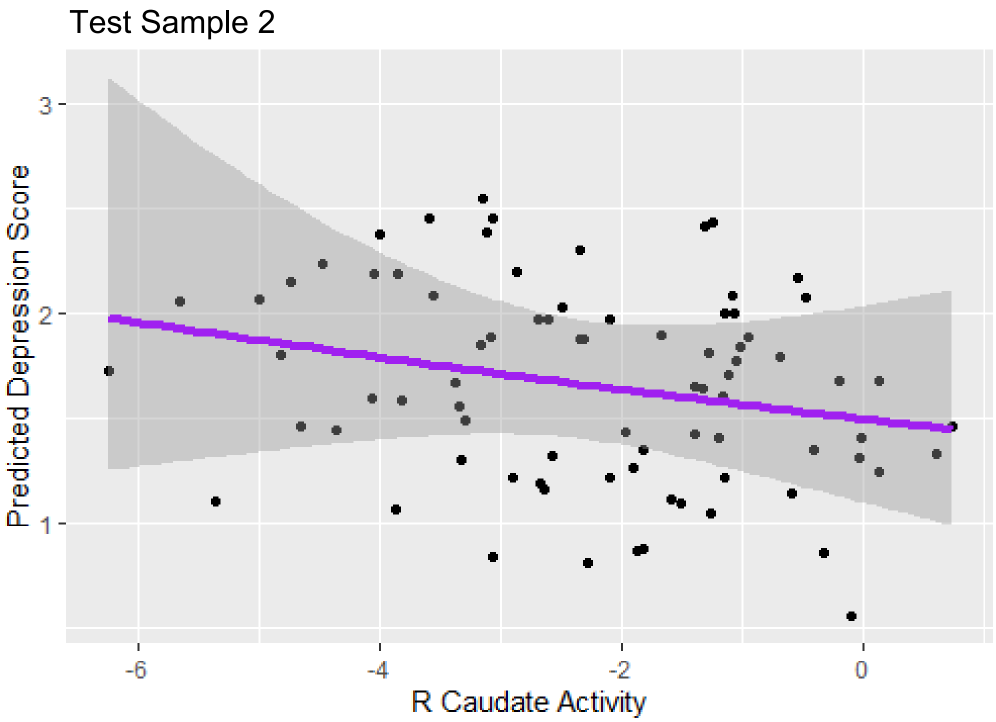

© 2023 Schumer MC et al. *JAMA Psychiatry.*

eTable 1. MOODS-SR manic and depressive mood domain descriptive statistics. A. Discovery sample; **B.** Test sample 1; **C.** Test sample 2. Abbreviations: MOODS-SR, Mood Spectrum Self-Report; SD, standard deviation; SE, standard error.

**A. Discovery sample (*n*=114)**						**Skewness**		**Kurtosis**	
	**Range**	**Minimum**	**Maximum**	**Mean**	**SD**	**Statistic**	**SE**	**Statistic**	**SE**
MOODS-SR manic mood domain	21	0	21	6.94	5.55	0.65	0.23	-0.35	0.45
MOODS-SR depressive mood domain	22	0	22	8.43	7.89	0.49	0.23	-1.40	0.45
**B. Test sample 1 (*n*=103)**						**Skewness**		**Kurtosis**	
	**Range**	**Minimum**	**Maximum**	**Mean**	**SD**	**Statistic**	**SE**	**Statistic**	**SE**
MOODS-SR manic mood domain	23	0	23	7.72	5.52	0.44	0.24	-0.39	0.47
MOODS-SR depressive mood domain	26	0	26	10.65	8.67	0.04	0.24	-1.66	0.47
**C. Test sample 2 (*n*=82)**						**Skewness**		**Kurtosis**	
	**Range**	**Minimum**	**Maximum**	**Mean**	**SD**	**Statistic**	**SE**	**Statistic**	**SE**
MOODS-SR manic mood domain	20	0	20	5.27	4.92	1.20	0.27	1.06	0.53
MOODS-SR depressive mood domain	25	0	25	6.45	7.21	1.06	0.27	-0.10	0.53

© 2023 Schumer MC et al. *JAMA Psychiatry.*

eTable 2. Significant activity and functional connectivity to approach-related facial emotions in the three samples. Abbreviations: dACC, dorsal anterior cingulate cortex; dlPFC, dorsolateral prefrontal cortex; FWE, family-wise error corrected; *k,* cluster size; L, left; MNI, Montreal Neuroimaging Institute; mPFC, medial prefrontal cortex; OFC, orbitofrontal cortex; R, right; vACC, ventral anterior cingulate cortex; vlPFC, ventrolateral prefrontal cortex.

**Discovery sample activation**					**Test sample 1 activation**					**Test sample 2 activation**				
**Region**	**MNI**	***k***	***t***	P_{FWE}	**Region**	**MNI**	***k***	***t***	P_{FWE}	**Region**	**MNI**	***k***	***t***	P_{FWE}
L amygdala	-20/-4/-14	88	10.82	.001	L amygdala	-22/-4/-18	60	6.60	.002					
R amygdala	22/-4/-14	653	9.87	<.001	R amygdala	26/-4/-14	66	6.30	.002					
L putamen	-22/6/0	762	10.79	<.001	L putamen	-22/-8/12	308	6.18	<.001					
R putamen	22/10/0	653	9.50	<.001										
L dlPFC[a]	-44/2/28	91	10.03	<.001										
R dlPFC	42/2/28	45	7.58	.003										
L dACC	-10/5/46	428	12.91	<.001	L dACC	-8/10/36	34	5.76	.005					
R dACC	10/12/40	245	8.62	<.001										
L insula	-42/-2/10	594	8.93	<.001	L insula	-42/-4/10	308	6.96	<.001					
L insula	-48/-24/20	151	8.97	<.001										
L insula	32/24/2	335	8.42	<.001										
R insula	56/-40/18	38	7.72	.004										
L vlPFC	-32/30/-2	21	6.45	.009										
R vlPFC[a]	32/30/-2	40	7.40	.003										
R OFC	26/42/-12	28	6.04	.006										
**Discovery sample deactivation**					**Test sample 1 deactivation**					**Test sample 2 deactivation**				
**Region**	**MNI**	***k***	***t***	P_{FWE}	**Region**	**MNI**	***k***	***t***	P_{FWE}	**Region**	**MNI**	***k***	***t***	P_{FWE}
R caudate[a]	4/8/6	355	16.86	<.001	R caudate[b]	4/8/6	2157	18.26	<.001	R caudate[b]	4/12/6	91	9.00	<.001
L caudate	-4/6/10	393	16.43	<.001	L caudate	-4/6/6	2157	17.85	<.001	L caudate	-4/10/8	84	9.55	<.001
R insula	44/-14/2	229	8.61	<.001	R insula	44/-14/-2	101	6.23	.001	R insula	44/-24/14	32	6.91	<.001
L insula	-44/-10/-8	120	9.54	<.001										
R dlPFC	6/52/38	56	7.25	.002	R dlPFC	26/42/38	24	6.81	.009					
L dlPFC	-2/40/38	83	7.26	.001	L dlPFC	-2/46/36	2157	9.61	<.001	L dlPFC	-2/56/21	23	7.02	<.001
L dlPFC	-20/36/40	28	6.18	.006	L dlPFC	-20/42/42	52	7.41	.003					
					L dlPFC	-28/32/42	45	6.60	.003					
					R mPFC	26/54/24	25	5.68	.008					
					L mPFC	-20/56/24	46	6.45	.003	L mPFC	-20/64/26	84	6.35	<.001
										L mPFC	-4/68/12	124	9.55	<.001

© 2023 Schumer MC et al. *JAMA Psychiatry.*

													L mPFC	-2/60/30	23	6.77	<.001
**Discovery sample functional connectivity**						**Test sample 1 functional connectivity**						**Test sample 2 functional connectivity**					
**Seed**	**Target**	**MNI**	***k***	***t***	**P_{FWE}**	**Seed**	**Target**	**MNI**	***k***	***t***	**P_{FWE}**	**Seed**	**Target**	**MNI**	***k***	***t***	**P_{FWE}**
amygdala	R amygdala	22/-4/-18	3502	33.51	<.001	amygdala	R amygdala	24/-4/-20	3893	27.80	<.001	amygdala	R amygdala	22/-2/-16	1994	14.87	<.001
	L amygdala[a]	-24/-4/-18	144	31.49	<.001		L amygdala[a]	-22/-6/-20	159	32.38	<.001		L amygdala[a]	-18/-6/-14	161	15.84	<.001
	R putamen	30/-4/4	3502	22.37	<.001		R putamen	22/8/-2	3893	26.48	<.001		R putamen	30/-10/6	1994	10.11	<.001
	L putamen	-22/4/4	3701	20.53	<.001		L putamen	-18/10/0	3830	25.55	<.001		L putamen	-26/-6/-8	2810	10.30	<.001
	L vACC	-10/-10/42	1424	14.90	<.001		L vACC	-2/-8/50	1579	15.96	<.001		L vACC	-10/-12/44	540	11.39	<.001
	R vACC	8/0/42	664	14.04	<.001		R vACC	4/-6/48	1413	16.37	<.001		R vACC	4/-4/50	417	10.67	<.001
	L dlPFC	-54/0/24	226	13.90	<.001		L dlPFC	-52/10/30	51	9.85	.002		L dlPFC	-52/10/32	313	9.74	<.001
	L dlPFC	-46/4/28	226	11.10	<.001		L dlPFC	-44/2/30	126	13.78	<.001		L dlPFC	-40/2/28	313	7.75	<.001
	R dlPFC	50/0/26	248	13.64	<.001		R dlPFC	52/2/36	129	16.97	<.001		R dlPFC	48/4/40	1059	11.67	<.001
	R dlPFC	52/10/30	248	11.34	<.001		R dlPFC	52/10/30	43	14.60	.003		R dlPFC	56/14/30	1059	10.24	<.001
	R dlPFC	46/30/12	253	12.89	<.001		R dlPFC	46/30/12	106	12.57	<.001						
							R dlPFC	32/38/30	33	7.48	.004						
							R dlPFC	24/38/40	27	6.76	.006		R dlPFC	20/40/36	41	6.96	<.001
	L dlPFC	-46/30/14	221	12.17	<.001		L dlPFC	-46/30/12	110	9.30	<.001		L dlPFC	-48/30/20	2810	10.26	<.001
	L dlPFC	-28/34/30	49	7.91	.002		L dlPFC	-30/38/30	45	7.55	.002						
	L dlPFC	-24/38/40	35	6.47	.004		L dlPFC	-26/40/40	52	6.95	.002						
	R OFC	4/42/-20	626	12.49	<.001		R OFC	4/50/-14	1413	8.88	<.001		R OFC	4/50/-10	832	8.80	<.001
	R OFC	24/26/-16	252	10.41	<.001												
	R vlPFC	36/30/-2	252	9.21	<.001								R vlPFC	36/32/-14	1059	11.43	<.001
													R vlPFC	26/16/-24	40	7.66	<.001
	L vlPFC	-48/34/-2	221	9.35	<.001								L vlPFC	-34/32/-14	2810	10.84	<.001
	L mPFC	-30/42/26	49	7.54	.002		L mPFC	-34/46/26	108	10.07	<.001		L mPFC	-32/44/26	53	6.12	<.001
							L mPFC	-38/50/16	20	8.31	.009						
							R mPFC	32/42/26	87	8.21	<.001						
													R mPFC	2/60/-4	832	10.62	<.001

© 2023 Schumer MC et al. *JAMA Psychiatry.*

Discovery sample functional connectivity						Test sample 1 functional connectivity						Test sample 2 functional connectivity					
**Seed**	**Target**	**MNI**	***k***	***t***	**P_{FWE}**	**Seed**	**Target**	**MNI**	***k***	***t***	**P_{FWE}**	**Seed**	**Target**	**MNI**	***k***	***t***	**P_{FWE}**
amygdala						amygdala						amygdala	L mPFC	-2/62/-4	944	10.39	<.001
							L caudate	-8/4/0	3830	16.04	<.001		L caudate	-12/-4/-16	393	8.13	<.001
	R caudate	8/4/-6	3502	15.50	<.001								R caudate	8/4/8	393	8.11	<.001
vlPFC	R vlPFC	48/28/-8	2852	20.39	<.001	vlPFC	R vlPFC	44/22/-12	1004	24.83	<.001	vlPFC	R vlPFC	48/44/-10	4751	18.60	<.001
	L vlPFC	-48/36/-4	2550	20.95	<.001		L vlPFC	-48/22/-10	1175	23.71	<.001		L vlPFC	-48/20/-8	6099	22.13	<.001
	R dlPFC	54/26/12	2852	22.99	<.001		R dlPFC	48/36/18	106	14.56	<.001		R dlPFC	56/24/0	4751	16.38	<.001
	R dlPFC[a]	44/6/40	398	17.20	<.001		R dlPFC[b]	46/8/40	243	15.13	<.001		R dlPFC[b]	54/18/28[c]	4751	16.46	<.001
	R dlPFC[a]	50/10/36	398	14.16	<.001		R dlPFC[b]	50/0/26	243	6.73	<.001						
							R dlPFC	50/16/26	43	16.79	.002						
	L dlPFC	-54/18/26	438	19.50	<.001		L dlPFC	-48/16/26	51	16.66	.001		L dlPFC	-52/20/26	6099	15.11	<.001
	L dlPFC	-44/6/40	438	15.73	<.001		L dlPFC	-46/8/38	283	16.16	<.001		L dlPFC	-44/6/40	6099	16.87	<.001
	L dlPFC	-48/28/18	2550	17.16	<.001		L dlPFC	-42/46/12	110	12.97	<.001						
	L dlPFC	-20/48/36	42	7.44	.002		L dlPFC	-16/48/36	47	8.06	.002						
	L dACC	-4/22/46	1630	16.75	<.001		L dACC	-2/24/42	1583	20.75	<.001		L dACC	-2/40/38	6099	16.46	<.001
	R dACC	4/22/46	1575	15.55	<.001		R dACC	4/22/46	1527	18.66	<.001						
	L putamen	-14/4/4	2550	18.09	<.001		L putamen	-14/8/0	1040	21.12	<.001						
							R putamen	16/6/-2	970	19.48	<.001						
	L insula	-48/42/20	46	7.26	.002		L insula	-48/-42/18	87	9.91	<.001						
							R insula	56/-40/20	95	15.46	<.001		R insula	56/-40/18	1479	10.61	<.001
							L mPFC	-44/46/0	110	14.36	<.001						
							L mPFC	-22/54/22	108	13.27	<.001						
							L mPFC	-38/48/14	20	13.06	.007						
							R mPFC	20/56/24	95	13.36	<.001		R mPFC	4/60/20	4751	15.85	<.001
							R mPFC	26/54/8	26	9.06	.005						
							L amygdala	-26/-2/-14	74	7.36	<.001						

© 2023 Schumer MC et al. *JAMA Psychiatry.*

**Discovery sample functional connectivity**						**Test sample 1 functional connectivity**						**Test sample 2 functional connectivity**					
**Seed**	**Target**	**MNI**	***k***	***t***	**P_{FWE}**	**Seed**	**Target**	**MNI**	***k***	***t***	**P_{FWE}**	**Seed**	**Target**	**MNI**	***k***	***t***	**P_{FWE}**
vlPFC						vlPFC	R amygdala	28/-2/-14	25	6.68	.005	vlPFC					
													R caudate	10/14/6	1479	13.63	<.001
dlPFC	L dlPFC	-40/22/34	460	24.05	<.001	dlPFC	L dlPFC	-44/14/40	269	21.07	<.001	dlPFC	L dlPFC	-48/20/38	5973	18.94	<.001
	L dlPFC	-18/36/42	50	13.34	.002		L dlPFC	-20/36/42	52	12.93	.001						
	L dlPFC	-2/36/38	1377	18.65	<.001		L dlPFC	-2/38/36	1015	23.02	<.001		L dlPFC	-2/20/46	5973	17.39	<.001
	L dlPFC	-46/20/28	460	17.74	<.001		L dlPFC	-46/18/28	51	19.38	.001		L dlPFC	-48/14/32	5973	17.44	<.001
	L dlPFC	-46/28/24	2506	18.25	<.001		L dlPFC	-46/28/22	110	16.96	<.001		L dlPFC	-46/32/20	5973	17.38	<.001
	R dlPFC[a]	42/14/34	467	21.84	<.001		R dlPFC[b]	42/16/40	232	20.43	<.001		R dlPFC[b]	48/6/40	6372	19.25	<.001
	R dlPFC	20/38/40	55	15.91	.002		R dlPFC	20/36/42	30	10.84	.004						
	R dlPFC	4/36/38	1339	19.23	<.001		R dlPFC	4/38/36	1003	22.22	<.001						
dlPFC	R dlPFC	48/32/20	2751	18.99	<.001	dlPFC	R dlPFC	48/32/22	106	18.81	<.001	dlPFC					
	R dlPFC	46/18/28	467	17.50	<.001		R dlPFC	50/22/26	43	18.71	.002		R dlPFC	52/16/32	6372	17.90	<.001
	L vlPFC	-32/18/-8	2506	20.72	<.001		L vlPFC	-28/18/-6	881	12.41	<.001						
	R vlPFC	32/20/-6	2751	19.43	<.001		R vlPFC	34/20/-10	646	12.68	<.001						
	R vlPFC	44/46/-14	2751	16.57	<.001		R vlPFC	46/40/-14	646	17.40	<.001		R vlPFC	50/44/-10	6372	16.82	<.001
	L dACC	-2/24/42	1377	20.71	<.001		L dACC	-2/24/42	1015	17.42	<.001		L dACC	-2/20/46	5973	17.39	<.001
	L dACC	-20/6/48	1377	11.47	<.001		L dACC	-20/6/48	42	7.67	.002						
	R dACC	4/24/42	1339	20.03	<.001		R dACC	4/24/42	1003	15.96	<.001		R dACC	4/22/44	6372	17.69	<.001
							R dACC	20/8/48	30	7.32	.004						
	L vACC	-2/-26/36	83	10.60	.001		L vACC	-2/-26/36	106	15.02	<.001						
	R vACC	4/-26/36	90	10.27	<.001		R vACC	4/-26/36	86	13.95	<.001						
	R insula	50/-42/20	42	10.47	.003		R insula	50/-42/20	46	11.87	.002						
	L amygdala	-28/-4/-14	29	9.92	.005												

© 2023 Schumer MC et al. *JAMA Psychiatry.*

Discovery sample functional connectivity						Test sample 1 functional connectivity						Test sample 2 functional connectivity					
**Seed**	**Target**	**MNI**	***k***	***t***	**P_{FWE}**	**Seed**	**Target**	**MNI**	***k***	***t***	**P_{FWE}**	**Seed**	**Target**	**MNI**	***k***	***t***	**P_{FWE}**
dlPFC	L mPFC	-20/52/26	131	9.59	<.001	dlPFC	L mPFC	-20/52/26	63	9.56	.001	dlPFC					
							L mPFC	-34/52/10	20	7.29	.008						
							R mPFC	20/56/24	95	12.05	<.001						
							R mPFC	24/56/4	31	9.13	.004						
	L OFC	-46/44/-10	2506	17.66	<.001		L OFC	-44/42/-12	881	20.76	<.001						
							R putamen	14/8/2	793	16.73	<.001						
							L putamen	-14/8/2	828	16.42	<.001						
dACC	L dACC	-2/34/28	1851	26.32	<.001	dACC	L dACC	-4/40/2	1823	25.04	<.001	dACC	L dACC	-2/28/32	3695	23.73	<.001
	R dACC	4/36/26	1760	24.54	<.001		R dACC	4/38/12	1726	27.38	<.001		R dACC	4/38/20	7329	22.43	<.001
	L putamen	-14/2/8	2059	20.26	<.001		L putamen	-14/10/-2	1771	18.84	<.001						
	R putamen	16/2/8	1925	16.68	<.001		R putamen	14/8/2	1667	18.01	<.001						
	L vlPFC	-28/16/-14	2059	20.07	<.001		L vlPFC	-28/18/-16	1771	16.57	<.001		L vlPFC	-28/30/-20	137	5.53	<.001
													L vlPFC	-46/16/-4	3078	16.29	<.001
	R vlPFC	30/18/-12	1925	16.43	<.001		R vlPFC	40/20/-14	1667	17.75	<.001		R vlPFC	48/16/-2	7329	18.15	<.001
	L mPFC	-22/44/26	178	17.59	<.001		L mPFC	-24/48/24	108	17.19	<.001						
	L mPFC[a]	-24/52/8	102	12.22	<.001		L mPFC[b]	-26/54/8	149	11.37	<.001		L mPFC[b]	-2/54/0[c]	3695	17.07	<.001
	L mPFC	-32/48/12	20	11.11	.009		L mPFC	-32/50/12	20	10.93	.006						
	L mPFC	-36/40/16	43	9.77	.003		L mPFC	-36/40/16	83	8.90	<.001						
	R mPFC	24/44/28	214	14.04	<.001		R mPFC	26/52/26	95	15.39	<.001		R mPFC	30/52/22	7329	18.66	<.001
	R mPFC	32/48/12	53	9.89	.002		R mPFC	42/40/24	59	8.79	.001						
	R mPFC	24/54/8	117	9.19	<.001		R mPFC	26/54/8	29	10.97	.003						
	L dlPFC	-26/32/34	382	15.07	<.001		L dlPFC	-26/32/38	257	12.48	<.001						
	L dlPFC[a]	-22/40/36	73	14.49	.001		L dlPFC[b]	-22/40/36	52	12.45	.001		L dlPFC[b]	-2/20/44[c]	3695	18.40	<.001
							L dlPFC	-48/10.36	51	8.26	.001						
	R dlPFC	28/32/34	324	13.11	<.001		R dlPFC	30/36/30	208	14.67	<.001						
	R dlPFC	24/40/36	69	12.17	.001		R dlPFC	26/42/36	30	12.72	.003		R dlPFC	30/48/30	7329	18.52	<.001

© 2023 Schumer MC et al. *JAMA Psychiatry.*

**Discovery sample functional connectivity**						**Test sample 1 functional connectivity**						**Test sample 2 functional connectivity**					
**Seed**	**Target**	**MNI**	***k***	***t***	**P_{FWE}**	**Seed**	**Target**	**MNI**	***k***	***t***	**P_{FWE}**	**Seed**	**Target**	**MNI**	***k***	***t***	**P_{FWE}**
dACC						dACC	R dlPFC	50/22/28	43	7.24	.002	dACC					
													L OFC	-28/40/-14	137	11.29	<.001
	L insula	-28/16/-6	2059	14.15	<.001		L insula	-28/18/-6	1771	13.03	<.001		L insula	-34/12/8	3078	13.19	<.001
							L insula	-30/-28/10	68	7.67	<.001		L insula	-32/-30/18	3078	14.00	<.001
							L insula	-48/-42/18	99	7.42	<.001		L insula	-48/-24/18	3078	14.44	<.001
							R insula	54/-40/20	54	8.77	.001						
							R insula	32/-24/10	58	6.58	.001						
	L caudate	-12/8/8	2059	14.31	<.001		L caudate	-10/6/4	1771	15.63	<.001		L caudate	-12/10/8	3078	14.56	<.001
	R caudate	12/4/12	1925	13.46	<.001												
putamen	R putamen	24/6/-2	3122	39.76	<.001	putamen	R putamen	24/8/-6	3439	38.57	<.001	putamen	R putamen	26/8/-4	7762	19.06	<.001
	L putamen	-22/6/-2	3149	38.72	<.001		L putamen	-18/8/-2	3253	31.03	<.001		L putamen	-22/10/-6	7144	18.55	<.001
	L amygdala	-26/-2/-14	115	30.66	<.001		L amygdala	-26/-2/-14	159	23.70	<.001		L amygdala	-22/-2/-14	25	12.11	<.001
													R amygdala	26/-2/-14	22	9.55	<.001
	R dACC	6/6/40	1054	18.37	<.001		R dACC	4/18/36	1476	16.54	<.001		R dACC	4/6/46	7762	16.73	<.001
	L dACC	-8/8/40	1090	19.66	<.001								L dACC	-2/8/48	7144	16.58	<.001
	L dACC[a]	-2/48/0	174	6.98	<.001		L dACC[b]	-4/12/44	1423	17.11	<.001		L dACC[b]	-6/28/28[c]	7144	17.77	<.001
	R mPFC[a]	8/44/-2	231	8.05	<.001								R mPFC[b]	30/52/26[c]	7762	17.42	<.001
	R mPFC	30/44/28	203	15.74	<.001		R mPFC	32/42/26	95	12.34	<.001		R mPFC	30/44/28	7762	16.29	<.001
	R mPFC	36/46/16	686	10.68	<.001		R mPFC	42/40/24	106	12.06	<.001						
	L mPFC	-30/42/26	163	14.20	<.001		L mPFC	-34/46/26	108	13.28	<.001						
	L mPFC	-36/40/16	220	13.01	<.001		L mPFC	-38/48/16	20	12.07	.006						
							L mPFC	-40/40/22	110	12.05	<.001						
							L mPFC	-40/50/4	89	9.80	<.001						
	R dlPFC	30/36/30	487	15.26	<.001		R dlPFC	30/38/30	260	11.71	<.001						

© 2023 Schumer MC et al. *JAMA Psychiatry.*

Discovery sample functional connectivity						Test sample 1 functional connectivity						Test sample 2 functional connectivity					
**Seed**	**Target**	**MNI**	***k***	***t***	**P_{FWE}**	**Seed**	**Target**	**MNI**	***k***	***t***	**P_{FWE}**	**Seed**	**Target**	**MNI**	***k***	***t***	**P_{FWE}**
putamen	R dlPFC	26/42/36	43	8.95	.002	putamen	R dlPFC	26/38/40	22	7.09	.006	putamen					
							R dlPFC	52/10/30	43	14.46	.002						
							R dlPFC	54/6/34	260	14.46	<.001						
	L dlPFC	-48/2/36	441	14.21	<.001		L dlPFC	-50/4/28	283	13.65	<.001						
							L dlPFC	-52/10/30	51	12.05	.001						
	L dlPFC	-24/42/36	42	9.41	.002		L dlPFC	-26/40/40	31	8.49	.003						
													L OFC	-28/40/-12	193	11.39	<.001
caudate	L caudate	-14/8/14	3833	30.36	<.001	caudate	L caudate	-10/14/-2	4729	25.90	<.001	caudate	L caudate	-10/0/14	7549	22.22	<.001
	R caudate	16/14/12	3833	29.53	<.001		R caudate	10/14/0	4729	25.12	<.001		R caudate	10/2/12	7889	20.34	<.001
	L vlPFC	-22/32/-6	177	20.26	<.001		L vlPFC	-22/32/-6	253	17.07	<.001						
	R vlPFC	22/30/-8	90	18.43	<.001		R vlPFC	24/32/-6	209	18.27	<.001						
	L dACC	-18/36/18	341	13.69	<.001												
	R dACC	20/38/16	345	13.81	<.001												
	L mPFC	-12/34/-10	214	10.94	<.001		L mPFC	-22/44/28	41	6.36	.003		L mPFC	-34/56/18	7549	14.12	<.001
	L amygdala	-20/-2/-16	112	10.21	<.001		L amygdala	-24/-2/-14	100	8.80	<.001						
	R amygdala	20/-2/-14	109	9.45	<.001		R amygdala	26/-2/-14	46	7.04	.003						
	L dlPFC	-28/34/30	36	7.96	.004		L dlPFC	-22/40/36	22	5.61	.008						
							L insula	32/-38/20	233	12.75	<.001						
							R putamen	30/-18/-8	48	8.21	.002						
	L putamen	-20/6/14	3833	21.66	<.001		L putamen	-20/6/14	4729	19.07	<.001		L putamen	-22/12/-6	7549	15.89	<.001

[a] Cluster identified in elastic-net variable selection models in the discovery sample.
[b] Cluster replicated in multivariable regression models in one or both of the independent test samples.
[c] Nearest peak and associated *t*-statistic are reported.

© 2023 Schumer MC et al. *JAMA Psychiatry.*

eTable 3. Additional mania/hypomania risk models. A. Discovery sample activity and functional connectivity related to MOODS-SR manic mood domain using all non-zero coefficients from both initial mania and depression risk models. **B.** Test sample 1 activity and functional connectivity related to MOODS-SR manic mood domain using all non-zero coefficients from both initial mania and depression risk models. **C.** Test sample 2 activity and functional connectivity related to MOODS-SR manic mood domain using all non-zero coefficients from both initial mania and depression risk models. Abbreviations: dACC, dorsal anterior cingulate cortex; dlPFC, dorsolateral prefrontal cortex; Exp, exponentiated; Exp(β), incidence rate ratio or a 1-unit change in independent variable is an Exp(β) increase in the dependent variable (mania risk score); L, left; mPFC, medial prefrontal cortex; N/A, not applicable; R, right; vlPFC, ventrolateral prefrontal cortex.

A. Discovery sample

Parameter	β	Hypothesis test		Exp (β)	Exp(β) 95% CI
		Wald χ^2	*P* value		
Age[c]	0.06	2.44	.119	1.06	0.98-1.14
L dlPFC	-0.09	21.18	<.001[a]	0.91	0.88-0.95
R vlPFC	-0.07	2.24	.134	0.93	0.85-1.02
R caudate[b]	-0.13	20.12	<.001[a]	0.88	0.83-0.93
amygdala-L amygdala[b]	0.34	80.71	<.001[a]	1.41	1.31-1.52
vlPFC-R dlPFC	0.03	2.77	.096	1.03	0.99-1.07
dACC-L mPFC	0.06	28.67	<.001[a]	1.07	1.04-1.09
dACC-L dlPFC	-0.02	3.31	.069	0.98	0.95-1.00
dlPFC-R dlPFC	0.11	28.54	<.001[a]	1.12	1.07-1.16
putamen-R MPFC	-0.01	0.29	.590	0.98	0.93-1.04
putamen-L dACC	0.01	0.35	.555	1.01	0.96-1.07

B. Test sample 1

Parameter	β	Hypothesis test		Exp (β)	Exp (β) 95% CI
		Wald χ^2	*P* value		
Age	0.05	1.91	0.167	1.05	0.98-1.13
L dlPFC	*N/A*	*N/A*	*N/A*	*N/A*	*N/A*
R vlPFC	*N/A*	*N/A*	*N/A*	*N/A*	*N/A*
R caudate[b]	-0.13	25.96	<.001[a]	0.88	0.83-0.92
amygdala-L amygdala[b]	0.43	126.92	<.001[a]	1.54	1.43-1.66
vlPFC-R dlPFC	0.10	9.95	.002[a]	1.10	1.04-1.17
dACC-L mPFC	0.01	0.11	.739	1.01	0.96-1.05
dACC-L dlPFC	0.01	0.37	.544	1.01	0.97-1.04
dlPFC-R dlPFC	-0.02	0.16	.685	0.98	0.91-1.06
putamen-R MPFC	*N/A*	*N/A*	*N/A*	*N/A*	*N/A*
putamen-L dACC	0.04	1.90	.168	1.04	0.98-1.09

C. Test sample 2

Parameter	β	Hypothesis test		Exp (β)	Exp(β) 95% CI
		Wald χ^2	*P* value		
Age	0.04	0.58	.447	1.04	0.93-1.16
L dlPFC	*N/A*	*N/A*	*N/A*	*N/A*	*N/A*
R vlPFC	*N/A*	*N/A*	*N/A*	*N/A*	*N/A*
R caudate[b]	-0.10	11.49	.001[a]	0.91	0.86-0.96
amygdala-L amygdala[b]	0.23	12.31	<.001[a]	1.25	1.10-1.42
vlPFC-R dlPFC	0.002	0.002	.964	1.00	0.94-1.07
dACC-L mPFC ext. to dlPFC	0.07	2.85	.091	1.07	0.99-1.17
dlPFC-R dlPFC	0.17	6.28	.012[a]	1.19	1.04-1.36
putamen-R MPFC	0.08	3.23	.072	1.09	0.99-1.19
putamen-L dACC	-0.09	3.08	.079	0.91	0.83-1.01

© 2023 Schumer MC et al. *JAMA Psychiatry.*

[a] Raw *P* values were significant at FDR-corrected thresholds; FDR *q* values are reported in the eResults.
[b] Finding replicated in all 3 samples.
[c] Age was *z*-scored for the multivariable regression analyses.

© 2023 Schumer MC et al. *JAMA Psychiatry.*

eTable 4. Additional depression risk models. A. Discovery sample activity and functional connectivity related to MOODS-SR depressive mood domain using all non-zero coefficients from both initial mania/hypomania and depression risk models. **B.** Test sample 1 activity and functional connectivity related to MOODS-SR depressive mood domain using all non-zero coefficients from both initial mania/hypomania and depression risk models. **C.** Test sample 2 activity and functional connectivity related to MOODS-SR depressive mood domain using all non-zero coefficients from both initial mania/hypomania and depression risk models. Abbreviations: dACC, dorsal anterior cingulate cortex; dlPFC, dorsolateral prefrontal cortex; Exp, exponentiated; Exp(β), incidence rate ratio or a 1-unit change in independent variable is an Exp(β) increase in the dependent variable (depression risk score); L, left; mPFC, medial prefrontal cortex; N/A, not applicable; R, right; vlPFC, ventrolateral prefrontal cortex.

**A. Discovery sample**						**B. Test sample 1**						**C. Test sample 2**					
**Parameter**	**β**	**Hypothesis test**		**Exp (β)**	**Exp(β) 95% CI**	**Parameter**	**β**	**Hypothesis test**		**Exp (β)**	**Exp(β) 95% CI**	**Parameter**	**β**	**Hypothesis test**		**Exp (β)**	**Exp (β) 95% CI**
		**Wald χ^2**	***P* value**					**Wald χ^2**	***P* value**					**Wald χ^2**	***P* value**		
Age[c]	-0.05	2.05	.152	0.95	0.89-1.02	Age	-0.02	0.32	.569	0.98	0.92-1.05	Age	0.24	22.89	<.001[a]	1.27	1.15-1.40
L dlPFC	-0.06	13.28	<.001[a]	0.94	0.90-0.97	L dlPFC	*N/A*	*N/A*	*N/A*	*N/A*	*N/A*	L dlPFC	*N/A*	*N/A*	*N/A*	*N/A*	*N/A*
R vlPFC	-0.07	3.19	.074	0.93	0.86-1.01	R vlPFC	*N/A*	*N/A*	*N/A*	*N/A*	*N/A*	R vlPFC	*N/A*	*N/A*	*N/A*	*N/A*	*N/A*
R caudate[b]	-0.18	50.90	<.001[a]	0.84	0.80-0.88	R caudate[b]	-0.11	24.38	<.001[a]	0.90	0.86-0.94	R caudate[b]	-0.10	13.36	<.001[a]	0.91	0.86-0.96
amygdala-L amygdala[b]	0.49	200.87	<.001[a]	1.63	1.52-1.74	amygdala-L amygdala[b]	0.31	74.32	<.001[a]	1.36	1.27-1.45	amygdala-L amygdala[b]	0.39	46.83	<.001[a]	1.48	1.32-1.66
vlPFC-R dlPFC	-0.02	1.97	.160	0.98	0.94-1.01	vlPFC-R dlPFC	0.09	10.66	.001[a]	1.09	1.04-1.15	vlPFC-R dlPFC	-0.01	0.05	.831	0.99	0.93-1.06
dACC-L mPFC	0.03	10.34	.001[a]	1.03	1.01-1.06	dACC-L mPFC	0.01	0.08	.776	1.01	0.97-1.05	dACC-L mPFC ext. to dlPFC	0.18	21.72	<.001[a]	1.19	1.10-1.29
dACC-L dlPFC	-0.07	38.55	<.001[a]	0.93	0.91-0.95	dACC-L dlPFC	0.01	0.41	.520	1.01	0.98-1.04						
dlPFC-R dlPFC	0.16	81.61	<.001[a]	1.17	1.13-1.21	dlPFC-R dlPFC	0.14	17.40	<.001[a]	1.16	1.08-1.24	dlPFC-R dlPFC	-0.06	0.89	.346	0.94	0.83-1.07
putamen-R MPFC	-0.02	0.69	.408	0.98	0.94-1.03	putamen-R MPFC	*N/A*	*N/A*	*N/A*	*N/A*	*N/A*	putamen-R MPFC	0.16	14.24	<.001[a]	1.18	1.08-1.28
putamen-L dACC	-0.03	2.04	.153	0.97	0.93-1.01	putamen-L dACC	0.14	34.06	<.001[a]	1.15	1.10-1.20	putamen-L dACC	-0.15	10.85	.001[a]	0.86	0.78-0.94

[a] Raw *P* values were significant at FDR-corrected thresholds; FDR *q* values are reported in the eResults.

© 2023 Schumer MC et al. *JAMA Psychiatry.*

[b] Finding replicated in all 3 samples.
[c] Age was *z*-scored for the multivariable regression analyses.

© 2023 Schumer MC et al. *JAMA Psychiatry.*

eTable 5. Post-hoc sensitivity analyses with mania/hypomania risk in unmedicated participants. A. Discovery sample activity and functional connectivity related to MOODS-SR manic mood domain after variable selection with elastic-net. **B.** Test sample 1 activity and functional connectivity related to MOODS-SR manic mood domain. Age was *z*-scored for the multivariable regression analyses. Abbreviations: dACC, dorsal anterior cingulate cortex; dlPFC, dorsolateral prefrontal cortex; Exp, exponentiated; Exp(β), incidence rate ratio or a 1-unit change in independent variable is an Exp(β) increase in the dependent variable (mania risk score); L, left; mPFC, medial prefrontal cortex; N/A, not applicable; R, right; vlPFC, ventrolateral prefrontal cortex.

**A. Discovery sample (*n*=113)**						**B. Test sample 1 (*n*=102)**					
**Parameter**	**β**	**Hypothesis test**		**Exp (β)**	**Exp(β) 95% CI**	**Parameter**	**β**	**Hypothesis test**		**Exp(β)**	**Exp(β) 95% CI**
		**Wald χ^2**	***P* value**					**Wald χ^2**	***P* value**		
Age[c]	0.02	0.26	.613	1.02	0.95-1.10	Age	0.05	2.06	.151	1.06	0.98-1.14
L dlPFC	-0.06	9.89	.002[a]	0.94	0.90-0.98	L dlPFC	*N/A*	*N/A*	*N/A*	*N/A*	*N/A*
R vlPFC	-0.13	7.94	.005[a]	0.87	0.80-0.96	R vlPFC	*N/A*	*N/A*	*N/A*	*N/A*	*N/A*
amygdala-L amygdala[b]	0.47	236.82	<.001[a]	1.59	1.50-1.69	amygdala-L amygdala[b]	0.50	431.86	<.001[a]	1.65	1.58-1.73
vlPFC-R dlPFC[b]	0.09	33.60	<.001[a]	1.10	1.06-1.13	vlPFC-R dlPFC[b]	0.10	13.77	<.001[a]	1.10	1.05-1.16
dACC-L mPFC	0.07	35.23	<.001[a]	1.07	1.05-1.10	dACC-L mPFC	0.02	1.11	.291	1.02	0.98-1.07

[a] Raw *P* values were significant at FDR-corrected thresholds; FDR *q* values are reported in the eResults.
[b] Finding replicated in all 3 samples.
[c] Age was *z*-scored for the multivariable regression analyses.

© 2023 Schumer MC et al. *JAMA Psychiatry.*

eTable 6. Post-hoc sensitivity analyses with depression risk in unmedicated participants. A. Discovery sample activity and functional connectivity related to MOODS-SR depressive mood domain after variable selection with elastic-net. **B.** Test sample 1 activity and functional connectivity related to MOODS-SR depressive mood domain. Abbreviations: dACC, dorsal anterior cingulate cortex; dlPFC, dorsolateral prefrontal cortex; Exp, exponentiated; Exp(β), incidence rate ratio or a 1-unit change in independent variable is an Exp(β) increase in the dependent variable (depression risk score); L, left; mPFC, medial prefrontal cortex; N/A, not applicable; r=right; vlPFC, ventrolateral prefrontal cortex.

**A. Discovery sample (*n*=113)**						**B. Test sample 1 (*n*=102)**					
**Parameter**	**β**	**Hypothesis test**		**Exp(β)**	**Exp(β) 95% CI**	**Parameter**	**β**	**Hypothesis test**		**Exp(β)**	**Exp(β) 95% CI**
		**Wald χ^2**	***P* value**					**Wald χ^2**	***P* value**		
Age[c]	-0.06	3.67	.055	0.94	0.88-1.00	Age	-0.04	1.67	.196	0.96	0.89-1.02
R vlPFC	-0.10	6.06	.014[a]	0.91	0.84-0.98	R vlPFC	*N/A*	*N/A*	*N/A*	*N/A*	*N/A*
R caudate[b]	-0.18	55.66	<.001[a]	0.84	0.80-0.88	R caudate[b]	-0.20	57.51	<.001[a]	0.82	0.78-0.86
amygdala-L amygdala[b]	0.49	229.003	<.001[a]	1.64	1.54-1.75	amygdala-L amygdala[b]	0.27	60.47	<.001[a]	1.31	1.23-1.41
dACC-L dlPFC	-0.06	28.19	<.001[a]	0.94	0.92-0.96	dACC-L dlPFC	0.01	0.78	.376	1.01	0.98-1.05
dlPFC-R dlPFC	0.13	73.56	<.001[a]	1.14	1.11-1.17	dlPFC-R dlPFC	0.21	52.16	<.001[a]	1.24	1.17-1.31
putamen-L dACC	-0.02	1.04	.308	0.98	0.94-1.02	putamen-L dACC	0.15	44.27	<.001[a]	1.17	1.11-1.22
putamen-R mPFC	-0.03	1.49	.222	0.97	0.93-1.02	putamen-R mPFC	*N/A*	*N/A*	*N/A*	*N/A*	*N/A*

[a] Raw *P* values were significant at FDR-corrected thresholds; FDR *q* values are reported in the eResults.
[b] Finding replicated in all 3 samples.
[c] Age was *z*-scored for the multivariable regression analyses.

© 2023 Schumer MC et al. *JAMA Psychiatry.*

eTable 7. Post-hoc sensitivity analyses with mania/hypomania risk in participants without MDD. A. Discovery sample activity and functional connectivity related to MOODS-SR manic mood domain after variable selection with elastic-net. **B.** Test sample 1 activity and functional connectivity related to MOODS-SR manic mood domain. **C.** Test sample 2 activity and functional connectivity related to MOODS-SR manic mood domain. Abbreviations: dACC, dorsal anterior cingulate cortex; dlPFC, dorsolateral prefrontal cortex; Exp, exponentiated; Exp(β), incidence rate ratio or a 1-unit change in independent variable is an Exp(β) increase in the dependent variable (mania risk score); L, left; MDD, Major Depressive Disorder; mPFC, medial prefrontal cortex; N/A, not applicable; R, right; vlPFC, ventrolateral prefrontal cortex.

**A. Discovery sample (*n*=86)**						**B. Test sample 1 (*n*=63)**						**C. Test sample 2 (*n*=71)**					
**Parameter**	**β**	**Hypothesis test**		**Exp (β)**	**Exp(β) 95% CI**	**Parameter**	**β**	**Hypothesis test**		**Exp (β)**	**Exp(β) 95% CI**	**Parameter**	**β**	**Hypothesis test**		**Exp (β)**	**Exp(β) 95% CI**
		**Wald χ^2**	***P* value**					**Wald χ^2**	***P* value**					**Wald χ^2**	***P* value**		
Age[c]	0.11	4.83	.028[a]	1.11	1.01-1.23	Age	0.11	4.42	.035[a]	1.12	1.01-1.24	Age	-0.03	0.19	.660	0.97	0.87-1.09
L dlPFC	-0.05	4.52	.033[a]	0.95	0.91-1.00	L dlPFC	*N/A*	*N/A*	*N/A*	*N/A*	*N/A*	L dlPFC	*N/A*	*N/A*	*N/A*	*N/A*	*N/A*
R vlPFC	-0.06	0.92	.337	0.94	0.83-1.07	R vlPFC	*N/A*	*N/A*	*N/A*	*N/A*	*N/A*	R vlPFC	*N/A*	*N/A*	*N/A*	*N/A*	*N/A*
amygdala-L amygdala[b]	0.40	97.44	<.001[a]	1.50	1.38-1.62	amygdala-L amygdala[b]	0.33	70.63	<.001[a]	1.39	1.29-1.50	amygdala-L amygdala[b]	0.32	29.49	<.001[a]	1.38	1.23-1.55
vlPFC-R dlPFC[b]	0.11	27.46	<.001[a]	1.11	1.07-1.16	vlPFC-R dlPFC[b]	0.19	20.69	<.001[a]	1.21	1.11-1.31	vlPFC-R dlPFC[b]	0.10	13.42	<.001[a]	1.10	1.05-1.16
dACC-L mPFC	0.06	14.71	<.001[a]	1.06	1.03-1.09	dACC-L mPFC	0.05	3.01	0.083	1.05	0.99-1.12	dACC-L mPFC	0.06	3.08	.079	1.07	0.99-1.15

[a] Raw *P* values were significant at FDR-corrected thresholds; FDR *q* values are reported in the eResults.
[b] Finding replicated in all 3 samples.
[c] Age was *z*-scored for the multivariable regression analyses.

© 2023 Schumer MC et al. *JAMA Psychiatry.*

eTable 8. Post-hoc sensitivity analyses with depression risk in participants without MDD. A. Discovery sample activity and functional connectivity related to MOODS-SR depressive mood domain after variable selection with elastic-net. **B.** Test sample 1 activity and functional connectivity related to MOODS-SR depressive mood domain. **C.** Test sample 2 activity and functional connectivity related to MOODS-SR depressive mood domain. Abbreviations: dACC, dorsal anterior cingulate cortex; dlPFC, dorsolateral prefrontal cortex; Exp, exponentiated; Exp(β), incidence rate ratio or a 1-unit change in independent variable is an Exp(β) increase in the dependent variable (depression risk score); L, left; MDD, Major Depressive Disorder; mPFC, medial prefrontal cortex; N/A, not applicable; R, right; vlPFC, ventrolateral prefrontal cortex.

**A. Discovery sample (*n*=86)**						**B. Test sample 1 (*n*=63)**						**C. Test sample 2 (*n*=71)**					
**Parameter**	**β**	**Hypothesis test**		**Exp (β)**	**Exp(β) 95% CI**	**Parameter**	**β**	**Hypothesis test**		**Exp (β)**	**Exp(β) 95% CI**	**Parameter**	**β**	**Hypothesis test**		**Exp (β)**	**Exp(β) 95% CI**
		**Wald X^2**	***P* value**					**Wald X^2**	***P* value**					**Wald X^2**	***P* value**		
Age[c]	-0.12	5.37	.020[a]	0.88	0.80-0.98	Age	0.05	0.60	.438	1.05	0.93-1.18	Age	0.19	12.29	<.001[a]	1.21	1.09-1.35
R vlPFC	-0.06	0.990	.320	0.94	0.82-1.06	R vlPFC	*N/A*	*N/A*	*N/A*	*N/A*	*N/A*	R vlPFC	*N/A*	*N/A*	*N/A*	*N/A*	*N/A*
R caudate[b]	-0.19	36.49	<.001[a]	0.82	0.77-0.88	R caudate[b]	-0.45	67.38	<.001[a]	0.63	0.57-0.71	R caudate[b]	-0.12	18.76	<.001[a]	0.89	0.84-0.94
amygdala-L amygdala[b]	0.48	93.00	<.001[a]	1.61	1.46-1.77	amygdala-L amygdala[b]	-0.17	6.94	.008[a]	0.84	0.74-0.96	amygdala-L amygdala[b]	0.39	42.84	<.001[a]	1.48	1.32-1.66
dACC-L dlPFC	-0.09	28.43	<.001[a]	0.91	0.88-0.94	dACC-L dlPFC	-0.01	0.11	.735	0.99	0.92-1.06	dACC-L dlPFC	0.13	10.79	.001[a]	1.14	1.05-1.23
dlPFC-R dlPFC	0.08	10.86	<.001[a]	1.08	1.03-1.14	dlPFC-R dlPFC	0.27	27.17	<.001[a]	1.31	1.18-1.45	dlPFC-R dlPFC	0.02	0.11	.738	1.02	0.91-1.15
putamen-L dACC	0.09	8.06	.005[a]	1.09	1.03-1.17	putamen-L dACC	0.19	23.25	<.001[a]	1.21	1.12-1.31	putamen-L dACC	-0.04	0.56	.455	0.96	0.88-1.06
putamen-R MPFC	-0.12	14.14	<.001[a]	0.87	0.83-0.94	putamen-R MPFC	*N/A*	*N/A*	*N/A*	*N/A*	*N/A*	putamen-R MPFC	0.02	0.29	.590	1.02	0.94-1.11

[a] Raw *P* values were significant at FDR-corrected thresholds; FDR *q* values are reported in the eResults.
[b] Finding replicated in all 3 samples.
[c] Age was *z*-scored for the multivariable regression analyses.

© 2023 Schumer MC et al. *JAMA Psychiatry.*

eTable 9. Post-hoc sensitivity analyses with mania/hypomania risk in participants without ADHD. A. Discovery sample activity and functional connectivity related to MOODS-SR manic mood domain after variable selection with elastic-net. **B.** Test sample 1 activity and functional connectivity related to MOODS-SR manic mood domain. **C.** Test sample 2 activity and functional connectivity related to MOODS-SR manic mood domain. Abbreviations: ADHD, Attention Deficit Hyperactivity Disorder; dACC, dorsal anterior cingulate cortex; dlPFC, dorsolateral prefrontal cortex; Exp, exponentiated; Exp(β), incidence rate ratio or a 1-unit change in independent variable is an Exp(β) increase in the dependent variable (mania risk score); L, left; mPFC, medial prefrontal cortex; N/A, not applicable; R, right; vlPFC, ventrolateral prefrontal cortex.

**A. Discovery sample (*n*=108)**						**B. Test sample 1 (*n*=101)**						**C. Test sample 2 (*n*=79)**					
**Parameter**	**β**	**Hypothesis test**		**Exp (β)**	**Exp(β) 95% CI**	**Parameter**	**β**	**Hypothesis test**		**Exp (*β*)**	**Exp(*β*) 95% CI**	**Parameter**	**β**	**Hypothesis test**		**Exp (β)**	**Exp(β) 95% CI**
		**Wald χ^2**	***P* value**					**Wald χ^2**	***P* value**					**Wald χ^2**	***P* value**		
Age[c]	0.06	2.76	.096	1.07	0.99-1.15	Age	0.07	3.92	.048	1.08	1.00-1.16	Age	0.05	0.89	.345	1.05	0.95-1.17
L dlPFC	-0.05	5.66	.017[a]	0.95	0.91-0.99	L dlPFC	*N/A*	*N/A*	*N/A*	*N/A*	*N/A*	L dlPFC	*N/A*	*N/A*	*N/A*	*N/A*	*N/A*
R vlPFC	-0.14	7.61	.006[a]	0.87	0.79-0.96	R vlPFC	*N/A*	*N/A*	*N/A*	*N/A*	*N/A*	R vlPFC	*N/A*	*N/A*	*N/A*	*N/A*	*N/A*
amygdala-L amygdala[b]	0.40	143.73	.000[a]	1.49	1.40-1.60	amygdala-L amygdala[b]	0.50	425.86	<.001[a]	1.65	1.57-1.73	amygdala-L amygdala[b]	0.33	35.68	<.001[a]	1.40	1.25-1.56
vlPFC-R dlPFC[b]	0.11	38.53	<.001[a]	1.11	1.08-1.15	vlPFC-R dlPFC[b]	0.10	16.17	<.001[a]	1.11	1.06-1.17	vlPFC-R dlPFC[b]	0.07	8.24	.004[a]	1.08	1.02-1.13
dACC-L mPFC	0.09	45.08	<.001[a]	1.09	1.06-1.12	dACC-L mPFC	0.02	0.81	.368	1.02	0.98-1.06	dACC-L mPFC	0.09	6.01	.014[a]	1.09	1.02-1.17

[a] Raw *P* values were significant at FDR-corrected thresholds; FDR *q* values are reported in the eResults.
[b] Finding replicated in all 3 samples.
[c] Age was *z*-scored for the multivariable regression analyses.

© 2023 Schumer MC et al. *JAMA Psychiatry.*

eTable 10. Post-hoc sensitivity analyses with depression risk in participants without ADHD. A. Discovery sample activity and functional connectivity related to MOODS-SR depressive mood domain after variable selection with elastic-net. **B.** Test sample 1 activity and functional connectivity related to MOODS-SR depressive mood domain. **C.** Test sample 2 activity and functional connectivity related to MOODS-SR depressive mood domain. Abbreviations: ADHD, Attention Deficit Hyperactivity Disorder; dACC, dorsal anterior cingulate cortex; dlPFC, dorsolateral prefrontal cortex; Exp, exponentiated; Exp(β), incidence rate ratio or a 1-unit change in independent variable is an Exp(β) increase in the dependent variable (depression risk score); L, left; mPFC, medial prefrontal cortex; N/A=not applicable; R, right; vlPFC, ventrolateral prefrontal cortex.

**A. Discovery sample (*n*=108)**						**B. Test sample 1 (*n*=101)**						**C. Test sample 2 (*n*=79)**					
**Parameter**	**β**	**Hypothesis test**		**Exp (β)**	**Exp(β) 95% CI**	**Parameter**	***β***	**Hypothesis test**		**Exp (β)**	**Exp(β) 95% CI**	**Parameter**	***β***	**Hypothesis test**		**Exp (β)**	**Exp(β) 95% CI**
		**Wald X^2**	***P* value**					**Wald X^2**	***P* value**					**Wald X^2**	***P* value**		
Age[c]	-0.04	1.65	.199	0.96	0.89-1.02	Age	-0.002	0.004	.951	1.10	0.93-1.06	Age	0.24	22.14	<.001[a]	1.27	1.15-1.40
R vlPFC	-0.12	8.14	.004[a]	0.89	0.82-0.96	R vlPFC	*N/A*	*N/A*	*N/A*	*N/A*	*N/A*	R vlPFC	*N/A*	*N/A*	*N/A*	*N/A*	*N/A*
R caudate[b]	-0.18	50.73	<.001[a]	0.83	0.79-0.87	R caudate[b]	-0.19	54.16	<.001[a]	0.82	0.78-0.87	R caudate[b]	-0.10	15.72	<.001[a]	0.90	0.85-0.95
amygdala-L amygdala[b]	0.45	181.33	<.001[a]	1.57	1.47-1.68	amygdala-L amygdala[b]	0.26	54.64	<.001[a]	1.30	1.21-1.39	amygdala-L amygdala[b]	0.40	50.56	<.001[a]	1.48	1.33-1.65
dACC-L dlPFC	-0.07	29.37	<.001[a]	0.93	0.91-0.96	dACC-L dlPFC	0.01	0.32	.571	1.01	0.98-1.04	dACC-L dlPFC	0.17	20.01	<.001[a]	1.18	1.10-1.28
dlPFC-R dlPFC	0.14	86.65	<.001[a]	1.15	1.12-1.19	dlPFC-R dlPFC	0.22	55.58	<.001[a]	1.25	1.18-1.32	dlPFC-R dlPFC	-0.07	1.53	.216	0.93	0.83-1.04
putamen-L dACC	-0.04	2.57	.109	0.96	0.92-1.01	putamen-L dACC	0.17	55.26	<.001[a]	1.19	1.13-1.24	putamen-L dACC	-0.16	10.73	.001[a]	0.85	0.78-0.94
putamen-R mPFC	-0.001	0.002	.96	1.00	0.95-1.05	putamen-R mPFC	*N/A*	*N/A*	*N/A*	*N/A*	*N/A*	putamen-R mPFC	0.17	16.45	<.001[a]	1.18	1.09-1.29

[a] Raw *P* values were significant at FDR-corrected thresholds; FDR *q* values are reported in the eResults.
[b] Finding replicated in all 3 samples.
[c] Age was *z*-scored for the multivariable regression analyses.

© 2023 Schumer MC et al. *JAMA Psychiatry.*

eTable 11. Significant activity and functional connectivity to all facial emotions in the three samples.
Abbreviations: dACC, dorsal anterior cingulate cortex; dlPFC, dorsolateral prefrontal cortex; FWE, family-wise error corrected; *k,* cluster size; L, left; MNI, Montreal Neuroimaging Institute; mPFC, medial prefrontal cortex; OFC, orbitofrontal cortex; R, right; vACC, ventral anterior cingulate cortex; vlPFC, ventrolateral prefrontal cortex.

**Discovery sample activation**					**Test sample 1 activation**					**Test sample 2 activation**				
**Region**	**MNI**	***k***	***t***	P_{FWE}	**Region**	**MNI**	***k***	***t***	P_{FWE}	**Region**	**MNI**	***k***	***t***	P_{FWE}
L dACC	-10/2/46	462	13.61	<.001	L dACC	-8/10/36	46	5.96	.003					
R dACC	10/4/46	293	10.24	<.001										
L putamen	-22/4/0	784	11.35	<.001	L putamen	-22/-8/12	93	5.84	.001					
R putamen	22/8/0	688	9.91	<.001										
L amygdala	-22/-4/-14	94	11.25	<.001	L amygdala	-22/-4/-18	49	6.29	.003					
R amygdala	20/-6/-14	688	10.46	<.001	R amygdala	26/-4/-14	54	6.06	.002					
L insula	-42/-2/10	681	10.08	<.001	L insula	-42/-4/8	209	7.03	<.001					
L insula	-48/-24/20	162	9.77	<.001	L insula	-52/-22/14	209	7.08	<.001					
R insula	32/22/2	485	8.63	<.001										
R insula	56/-40/18	45	8.07	.003										
L dlPFC	-44/2/28	85	9.73	.001										
R dlPFC	42/2/28	47	7.85	.002										
R vlPFC[a]	32/30/-2	80	7.39	.001										
L vlPFC	-26/16/-12	7.45	681	<.001										
**Discovery sample deactivation**					**Test sample 1 deactivation**					**Test sample 2 deactivation**				
**Region**	**MNI**	***k***	***t***	P_{FWE}	**Region**	**MNI**	***k***	***t***	P_{FWE}	**Region**	**MNI**	***k***	***t***	P_{FWE}
R caudate[a]	4/6/6	368	17.59	<.001	R caudate[b]	4/8/6	1764	18.62	<.001	R caudate[b]	6/6/10	89	10.37	<.001
L caudate	-4/6/8	414	16.49	<.001	L caudate	-4/6/6	1764	18.17	<.001	L caudate	-4/10/6	61	9.57	<.001
L insula	-42/-16/-6	116	9.12	<.001										
R insula	44/-14/0	235	8.67	<.001	R insula	40/-24/4	106	6.40	<.001					
R dlPFC[a]	6/54/38	67	7.31	.001	R dlPFC[b]	4/44/36	428	9.07	<.001					
					R dlPFC	26/42/38	24	6.87	.008					
L dPFC	-2/38/38	114	7.11	<.001	L dlPFC	-16/48/38	52	8.23	.002					
L dlPFC	-22/38/38	37	6.59	.004	L dlPFC	-28/32/42	53	7.02	.002					
L vlPFC	-54/34/-4	25	7.31	.007	L vlPFC	-34/14/-20	21	10.14	.010					
					L mPFC	-20/56/24	48	6.83	.003	L mPFC	-24/66/14	83	7.18	<.001
										L mPFC	-2/58/-2	77	8.05	<.001

© 2023 Schumer MC et al. *JAMA Psychiatry.*

						R mPFC		26/54/24	25	5.87	.008						
**Discovery sample functional connectivity**						**Test sample 1 functional connectivity**						**Test sample 2 functional connectivity**					
**Seed**	**Target**	**MNI**	***k***	***t***	**P_{FWE}**	**Seed**	**Target**	**MNI**	***k***	***t***	**P_{FWE}**	**Seed**	**Target**	**MNI**	***k***	***t***	**P_{FWE}**
amygdala	R amygdala	24/-4/-18	3551	35.52	<.001	amygdala	R amygdala	24/-4/-20	3928	30.06	<.001	amygdala	R amygdala	20/-2/-16	3523	18.68	<.001
	L amygdala[a]	-24/-4/-18	144	33.26	<.001		L amygdala[a]	-24-6/-18	259	34.93	<.001		L amygdala[a]	-22/-2/-16	161	18.04	<.001
	R putamen	30/-2/6	3551	25.90	<.001		R putamen	30/-6/-8	3928	27.63	<.001						
	L putamen	-26/10/2	4007	23.03	<.001		L putamen	-20/8/-4	3880	28.65	<.001		L putamen	-30/-20/8	4989	11.50	<.001
	L dlPFC	-54/0/24	228	16.46	<.001		L dlPFC	-54/0/26	122	15.81	<.001		L dlPFC	-56/6/30	360	10.57	<.001
	L dlPFC	-52/10/30	228	11.64	<.001		L dlPFC	-52/10/30	51	11.12	.002		L dlPFC	-52/10/32	360	10.37	<.001
							L dlPFC	-46/30/12	110	10.49	<.001						
	L dlPFC	-28/36/30	55	9.23	.002		L dlPFC	-30/38/30	50	7.73	.002						
	L dlPFC	-24/38/40	38	6.91	.003		L dlPFC	-24/40/32	52	8.23	.001						
													L dlPFC	-42/32/40	20	5.77	<.001
	R dlPFC	54/2/34	246	15.72	<.001		R dlPFC	52/2/36	128	18.90	<.001		R dlPFC	54/4/40	3523	11.80	<.001
	R dlPFC	48/32/14	277	14.61	<.001		R dlPFC	46/30/12	106	13.67	<.001						
	R dlPFC	32/42/30	50	6.72	.002		R dlPFC	28/32/38	35	7.92	.003		R dlPFC	26/38/40	42	7.06	<.001
							R dlPFC	50/10/30	43	16.99	.002		R dlPFC	56/14/32	3523	11.50	<.001
							R dlPFC	24/38/40	30	7.66	.004		R dlPFC	20/40/38	42	7.75	<.001
	L vACC	-8/-10/44	765	16.33	<.001		L vACC	-2/-8/50	1640	17.77	<.001		L vACC	-2/-4/50	609	12.22	
	R vACC	8/0/42	684	15.87	<.001		R vACC	4/-6/48	1441	18.21	<.001		R vACC	4/-4/44	493	12.87	<.001
	R OFC	4/42/-20	595	13.95	<.001		R OFC	4/42/-20	1441	9.14	<.001		R OFC	4/60/-10	792	11.39	<.001
	L OFC	-4/48/-14	652	13.40	<.001		L OFC	-4/50/-16	1640	9.35	<.001						
	R vlPFC	24/22/-18	270	11.46	<.001		R vlPFC	22/10/-18	3928	18.73	<.001		R vlPFC	36/36/-14	3523	13.22	<.001
							L vlPFC	-22/10/-18	3880	18.84	<.001		L vlPFC	-34/32/-14	4989	12.11	<.001
	L mPFC	-30/42/28	84	8.87	<.001		L mPFC	-34/46/26	104	9.60	<.001						
							L mPFC	-36/46/16	20	8.02	.008						
							R mPFC	32/42/26	83	7.54	<.001						
	L insula	-34/-8/10	4007	16.44	<.001		L insula	-34/-10/10	3880	15.93	<.001		L insula	-32/-24/10	4989	12.93	<.001
							R insula	38/-6/-8	3928	17.37	<.001		R insula	38/-4/12	3523	11.89	<.001

© 2023 Schumer MC et al. *JAMA Psychiatry.*

Discovery sample functional connectivity						Test sample 1 functional connectivity						Test sample 2 functional connectivity					
**Seed**	**Target**	**MNI**	***k***	***t***	**P_{FWE}**	**Seed**	**Target**	**MNI**	***k***	***t***	**P_{FWE}**	**Seed**	**Target**	**MNI**	***k***	***t***	**P_{FWE}**
vlPFC	R dlPFC	54/26/12	3208	24.99	<.001	vlPFC	R dlPFC	52/28/18	106	16.15	<.001	vlPFC	R dlPFC	54/24/0	6813	17.53	<.001
	R dlPFC[a]	46/8/40	416	18.13	<.001		R dlPFC[b]	44/10/40	247	16.30	<.001		R dlPFC[b]	48/4/40	6813	17.19	<.001
	R dlPFC	18/48/36	43	9.08	.002												
							R dlPFC	50/16/26	43	17.78	.002						
	L dlPFC	-52/18/26	464	20.98	<.001		L dlPFC	-48/16/26	51	19.10	.001						
	L dlPFC	-46/6/40	464	16.64	<.001		L dlPFC	-46/8/38	282	16.22	<.001		L dlPFC	-44/6/40	6538	16.68	<.001
	L dlPFC	-48/28/18	2729	18.03	<.001		L dlPFC	-48/28/18	110	14.10	<.001		L dlPFC	-56/28/16	6538	17.03	<.001
	L dlPFC	-16/48/36	60	7.68	.001		L dlPFC	-16/48/36	37	8.15	.003						
	R vlPFC	52/24/2	3208	23.41	<.001		R vlPFC	46/24/-12	1003	26.12	<.001		R vlPFC	52/18/-2	6813	18.54	<.001
	L vlPFC	-48/36/-2	2729	21.38	<.001		L vlPFC	-48/22/-10	1149	25.43	<.001		L vlPFC	-48/20/-8	6538	22.69	<.001
	L dACC	-4/22/46	1732	18.81	<.001		L dACC	-2/24/42	1581	22.36	<.001						
	R dACC	6/22/46	1704	17.83	<.001		R dACC	4/22/46	1548	19.62	<.001		R dACC	4/22/44	6813	17.66	<.001
	L insula	-48/-42/20	63	7.94	.001		L insula	-48/-42/18	1144	10.95	<.001						
							R insula	54/-40/18	109	16.07	<.001						
							R putamen	14/8/0	1014	23.08	<.001						
							L putamen	-14/8/0	1144	22.69	<.001						
							L mPFC	-44/46/0	110	14.96	<.001						
	L mPFC	-22/48/22	1732	13.87	<.001		L mPFC	-22/54/22	108	14.22	<.001						
							L mPFC	-38/46/14	20	13.47	.007						
							R mPFC	22/54/26	95	14.51	<.001						
							R mPFC	28/54/8	27	9.96	.005						
							R amygdala	28/-4/-14	22	7.57	.006						
							L amygdala	-26/-2/-14	41	7.31	.002						

© 2023 Schumer MC et al. *JAMA Psychiatry.*

Discovery sample functional connectivity						Test sample 1 functional connectivity						Test sample 2 functional connectivity					
**Seed**	**Target**	**MNI**	***k***	***t***	**P_{FWE}**	**Seed**	**Target**	**MNI**	***k***	***t***	**P_{FWE}**	**Seed**	**Target**	**MNI**	***k***	***t***	**P_{FWE}**
dlPFC	L dlPFC	-42/20/34	469	26.34	<.001	dlPFC	L dlPFC	-44/16/40	253	22.23	<.001	dlPFC	L dlPFC	-48/20/38	6183	19.61	<.001
	L dlPFC	-46/18/28	469	22.95	<.001		L dlPFC	-46/18/28	51	19.41	.001		L dlPFC	-48/14/32	6183	18.83	<.001
							L dlPFC	-46/28/24	110	18.52	<.001						
	L dlPFC	-18/36/42	53	14.43	.002		L dlPFC	-18/36/42	52	12.96	.001						
	R dlPFC[a]	42/14/34	481	23.53	<.001		R dlPFC[a]	44/16/40	232	22.94	<.001		R dlPFC[b]	46/10/30	6632	22.33	<.001
	R dlPFC	54/28/12	2873	22.55	<.001		R dlPFC	50/20/26	43	20.27	.002		R dlPFC	50/30/24	6632	18.25	<.001
	R dlPFC	48/34/20	2873	18.80	<.001		R dlPFC	48/32/22	106	19.25	<.001		R dlPFC	46/30/36	6632	19.21	<.001
	R dlPFC	20/40/38	60	20.26	.001		R dlPFC	20/38/42	30	11.38	.004						
	L dACC	-4/24/40	1449	23.82	<.001		L dACC	-4/22/46	924	17.92	<.001		L dACC	-4/14/46	6183	18.90	<.001
	L dACC	-10/10/48	1449	15.79	<.001		L dACC	-10/10/50	43	7.56	.002						
	R dACC	4/24/40	1569	22.48	<.001		R dACC	4/24/42	949	16.62	<.001		R dACC	4/22/44	6632	19.71	<.001
							R dACC	14/10/52	28	6.92	.004						
	L vACC	-2/-26/36	127	12.59	<.001		L vACC	-2/-26/36	90	14.55	<.001						
							R vACC	4/-26/36	59	13.75	.001						
	L vlPFC	-44/40/-12	2582	20.67	<.001		L vlPFC	-46/42/-12	852	20.08	<.001						
	R vlPFC	44/38/-10	2873	21.74	<.001		R vlPFC	44/36/-14	647	18.32	<.001						
	R insula[a]	50/-42/20	50	12.27	50		R insula[b]	50/-42/20	48	11.70	.001						
	L amygdala	-28/-4/-14	33	11.77	.004												
	L mPFC	-20/52/26	124	10.39	<.001		L mPFC	-20/52/26	58	10.50	.001						
							R mPFC	20/56/24	67	13.13	.001						
							R mPFC	38/40/22	21	9.77	.006		R mPFC	30/54/18	6632	17.52	<.001
							R mPFC	24/56/4	28	8.70	.004						
							R putamen	14/8/2	801	18.42	<.001						

© 2023 Schumer MC et al. *JAMA Psychiatry.*

Discovery sample functional connectivity						Test sample 1 functional connectivity						Test sample 2 functional connectivity					
**Seed**	**Target**	**MNI**	***k***	***t***	**P_{FWE}**	**Seed**	**Target**	**MNI**	***k***	***t***	**P_{FWE}**	**Seed**	**Target**	**MNI**	***k***	***t***	**P_{FWE}**
dlPFC						dlPFC	L putamen	-14/8/2	785	17.55	<.001	dlPFC					
dACC	L dACC	-4/34/28	1889	25.99	<.001	dACC	L dACC	-2/42/2	1827	27.02	<.001	dACC	L dACC	-2/28/32	3794	25.65	<.001
	R dACC	4/36/28	2045	25.48	<.001		R dACC	2/42/2	1740	27.08	<.001		R dACC	4/24/38	7613	24.05	<.001
	L putamen	-14/4/6	2099	22.57			L putamen	-12/6/-4	1776	20.00	<.001						
	L vlPFC	-28/16/-14	2099	22.41	<.001		L vlPFC	-28/8/-16	1776	19.11	<.001		L vlPFC	-28/40/-14	157	11.71	<.001
	R vlPFC[a]	30/18/-12	2050	20.06	<.001		R vlPFC[a]	40/20/-16	1678	18.16	<.001		R vlPFC[b]	30/44/26[c]	7613	19.50	<.001
	R putamen[a]	16/2/8	2050	18.39	<.001		R putamen[a]	14/8/-2	1678	20.49	<.001						
	L mPFC	-22/44/28	183	20.28	<.001		L mPFC	-24/50/22	108	19.51	<.001						
	L mPFC	-24/52/8	112	13.66	<.001		L mPFC	-26/54/8	133	12.97	<.001						
	L mPFC	-36/40/16	36	9.85	.003		L mPFC	-32/50/12	20	10.85	.006						
							L mPFC	-40/40/22	53	8.07	.001						
	R mPFC	26/58/8	140	10.18	<.001		R mPFC	26/54/8	29	12.15	.003						
							R mPFC	28/54/20	95	15.58	<.001						
	R mPFC	32/48/12	51	9.96	.002		R mPFC	38/40/16	40	7.30	.002						
	L dlPFC	-26/32/34	398	16.91	<.001		L dlPFC	-28/36/30	245	12.46	<.001						
	L dlPFC[a]	-22/40/36	73	16.70	.001		L dlPFC[b]	-24/42/36	52	12.87	.001		L dlPFC[b]	-2/20/44[c]	3794	19.97	<.001
	L dlPFC	-48/10/36	398	7.80	<.001		L dlPFC	-48/10/36	50	7.01	.001						
	R dlPFC	28/30/34	326	14.59	<.001		R dlPFC	30/36/30	212	15.00	<.001						
	R dlPFC	22/38/38	69	14.16	.001		R dlPFC	26/44/36	30	14.21	.003						
							R dlPFC	50/10/36	43	7.59	.001						
							R insula	38/-28/14	163	6.87	<.001						
							L insula	-38/-28/14	167	7.97	<.001		L insula	-32/-30/18	3255	15.05	<.001
	L insula	-28/16/-6	2099	14.32	<.001								L insula	-34/12/8	3255	15.63	<.001
putamen	L putamen	-22/6/-2	3233	45.09	<.001	putamen	L putamen	-28/-14/0	34.46	3270	<.001	putamen	L putamen	-30/-18/2	7378	20.80	<.001

© 2023 Schumer MC et al. *JAMA Psychiatry.*

Discovery sample functional connectivity						Test sample 1 functional connectivity						Test sample 2 functional connectivity					
**Seed**	**Target**	**MNI**	***k***	***t***	**P_{FWE}**	**Seed**	**Target**	**MNI**	***k***	***t***	**P_{FWE}**	**Seed**	**Target**	**MNI**	***k***	***t***	**P_{FWE}**
putamen	R putamen	26/6/-4	3222	44.85	<.001	putamen	R putamen	26/0/0	3431	40.70	<.001	putamen	R putamen	28/0/-6	8012	21.25	<.001
	L amygdala	-26/-2/-14	116	30.63	<.001		L amygdala	-26/-2/-14	159	24.25	<.001		L amygdala	-22/-2/-14	41	13.35	<.001
	R amygdala	28/-2/-14	3222	27.89	<.001		R amygdala	28/-2/-14	3431	22.53	<.001		R amygdala	28/-2/-14	24	11.91	<.001
	L vACC	-8/4/38	1040	22.18	<.001		L vACC	-2/0/50	1231	18.25	<.001		L vACC	-8/10/36	7378	21.30	<.001
	R dACC	8/10/40	1030	20.32	<.001		R dACC	6/16/36	1263	17.69	<.001		R dACC	8/28/28	8012	20.43	<.001
	L dACC[a]	-8/44/-2	134	7.29	<.001								L dACC[b]	-6/28/28[c]	7378	20.30	<.001
													L dACC	-2/8/48	7378	21.54	<.001
	R dlPFC	52/4/32	497	17.48	<.001		R dlPFC	54/4/32	257	16.25	<.001						
							R dlPFC	52/10/30	43	15.97	.002						
	R dlPFC	26/42/36	49	9.95	.001												
	L dlPFC	-28/36/30	450	16.73	<.001		L dlPFC	-30/38/30	267	14.92	<.001						
	L dlPFC	-24/42/36	39	9.40	.002		L dlPFC	-26/40/40	25	8.51	.004						
							L dlPFC	-52/10/30	51	13.35	.001						
	R mPFC	30/44/28	1031	17.08	<.001		R mPFC	34/42/24	95	12.98	<.001		R mPFC	30/44/28	8012	19.41	<.001
							R mPFC	42/40/24	106	12.54	<.001						
	L mPFC	-30/42/26	158	16.75	<.001		L mPFC	-34/46/26	106	13.90	<.001						
	L mPFC	-36/40/16	282	15.13	<.001		L mPFC	-38/48/16	20	12.45	.006						
							L mPFC	-40/40/22	110	12.29	<.001						
	L mPFC	-34/60/8	22	6.41	.006		L mPFC	-30/54/8	90	9.77	<.001						
	R OFC[a]	10/40/-6	177	8.89	<.001								R OFC[b]	8/28/28[c]	8012	20.43	<.001
													L OFC	-28/40/-14	213	12.58	<.001
caudate	L caudate[a]	-14/8/12	3901	33.24	<.001	caudate	L caudate[b]	-10/14/-2	5797	30.64	<.001	caudate	L caudate[b]	-10/0/14	8075	26.24	<.001
	R caudate	14/14/10	3901	31.97	<.001		R caudate	12/14/-2	5797	27.95	<.001		R caudate	10/2/12	8187	21.71	<.001
	L vlPFC	-22/32/-6	172	21.15	<.001		L vlPFC	-22/30/-8	320	18.33	<.001						

© 2023 Schumer MC et al. *JAMA Psychiatry.*

Discovery sample functional connectivity						Test sample 1 functional connectivity						Test sample 2 functional connectivity					
**Seed**	**Target**	**MNI**	***k***	***t***	**P_{FWE}**	**Seed**	**Target**	**MNI**	***k***	***t***	**P_{FWE}**	**Seed**	**Target**	**MNI**	***k***	***t***	**P_{FWE}**
caudate	R vlPFC	24/32/-6	102	20.66	<.001	caudate	R vlPFC	24/32/-6	254	17.75	<.001	caudate					
	R dACC	20/38/16	179	15.93	<.001												
	R dACC	16/16/34	176	9.27	<.001								R dACC	10/34/20	8187	17.93	<.001
	L dACC	-18/36/18	344	15.29	<.001								L dACC	-4/34/20	8075	15.75	<.001
	L amygdala	-20/-2/-16	110	10.33	<.001		L amygdala	-24/-2/-14	107	10.51	<.001						
	R amygdala	22/-2/-14	93	8.60	<.001												
	L mPFC	-12/36/-10	218	10.15	<.001												
							L mPFC	-22/44/30	44	6.63	.003		L mPFC	-32/56/18	8075	16.01	<.001
							R mPFC	24/44/28	44	7.07	.003						
	L dlPFC	-28/34/30	25	7.76	.006		L dlPFC	-28/36/34	20	5.81	.009						
							L dlPFC	-24/42/36	44	7.49	.003						
							R dlPFC	28/30/34	27	6.76	.006						
							R dlPFC	26/42/36	23	6.11	.007						
							R insula	32/-38/20	294	11.04	<.001						
							L vACC	-14/-8/46	22	6.86	.008						

[a] Cluster identified in elastic-net variable selection models in the discovery sample.
[b] Cluster replicated in multivariable regression models in one or both of the independent test samples.
[c] Nearest peak and associated *t*-statistic are reported.

© 2023 Schumer MC et al. *JAMA Psychiatry.*

eTable 12. All facial emotions analysis results for mania/hypomania risk. A. Discovery sample activity and functional connectivity related to MOODS-SR manic mood domain after variable selection with elastic-net. **B.** Test sample 1 activity and functional connectivity related to MOODS-SR manic mood domain. **C.** Test sample 2 activity and functional connectivity related to MOODS-SR manic mood domain. Abbreviations: dACC, dorsal anterior cingulate cortex; dlPFC, dorsolateral prefrontal cortex; Exp, exponentiated; Exp(β), incidence rate ratio or a 1-unit change in independent variable is an Exp(β) increase in the dependent variable (mania risk score); L, left; mPFC, medial prefrontal cortex; N/A, not applicable; R, right; vlPFC, ventrolateral prefrontal cortex.

**A. Discovery sample**						**B. Test sample 1**						**C. Test sample 2**					
**Parameter**	**β**	**Hypothesis test**		**Exp (β)**	**Exp(β) 95% CI**	**Parameter**	**β**	**Hypothesis test**		**Exp (β)**	**Exp(β) 95% CI**	**Parameter**	**β**	**Hypothesis test**		**Exp (β)**	**Exp(β) 95% CI**
		**Wald χ^2**	***P* value**					**Wald χ^2**	***P* value**					**Wald χ^2**	***P* value**		
Age[b]	0.06	81.90	<.001[a]	1.06	1.05-1.08	Age	0.07	166.87	<.001[a]	1.07	1.06-1.09	Age	0.07	107.52	<.001[a]	1.07	1.06-1.08
R vlPFC	-0.37	14.47	<.001[a]	0.69	0.57-0.84	R vlPFC	*N/A*	*N/A*	*N/A*	*N/A*	*N/A*	R vlPFC	*N/A*	*N/A*	*N/A*	*N/A*	*N/A*
R dlPFC	-0.08	10.52	.001[a]	0.92	0.88-0.97	R dlPFC	0.002	0.002	.968	1.00	0.89-1.13	R dlPFC	*N/A*	*N/A*	*N/A*	*N/A*	*N/A*
caudate-L caudate	0.09	0.77	.381	1.10	0.89-1.36	caudate-L caudate	0.06	0.64	.422	1.07	0.91-1.25	caudate-L caudate	0.18	13.24	<.001[a]	1.20	1.09-1.33
dACC-R vlPFC ext. to putamen	0.30	14.35	<.001[a]	1.35	1.15-1.57	dACC-R vlPFC ext. to putamen	0.18	8.00	.005[a]	1.19	1.06-1.35	dACC-R vlPFC ext. to putamen	-0.01	0.01	.935	0.99	0.80-1.23
dlPFC-R insula	0.18	12.80	<.001[a]	1.20	1.09-1.33	dlPFC-R insula	-0.06	1.46	.227	0.94	0.85-1.04	dlPFC-R insula	*N/A*	*N/A*	*N/A*	*N/A*	*N/A*
vlPFC-R dlPFC	0.04	1.08	.298	1.04	0.97-1.12	vlPFC-R dlPFC	0.20	11.31	.001[a]	1.22	1.08-1.36	vlPFC-R dlPFC	-0.15	3.30	.069	0.86	0.73-1.01

[a] Raw *P*-values were significant at FDR-corrected thresholds; FDR *q*-values are reported in the eResults.
[b] Age was *z*-scored for the multivariable regression analyses.

© 2023 Schumer MC et al. *JAMA Psychiatry.*

eTable 13. All facial emotions analysis results for depression risk. A. Discovery sample activity and functional connectivity related to MOODS-SR depressive mood domain after variable selection with elastic-net. **B.** Test sample 1 activity and functional connectivity related to MOODS-SR depressive mood domain. **C.** Test sample 2 activity and functional connectivity related to MOODS-SR depressive mood domain. Abbreviations: dACC, dorsal anterior cingulate cortex; dlPFC, dorsolateral prefrontal cortex; Exp, exponentiated; Exp(β), incidence rate ratio or a 1-unit change in independent variable is an Exp(β) increase in the dependent variable (depression risk score); L, left; mPFC, medial prefrontal cortex; N/A, not applicable; R, right; OFC, orbitofrontal cortex; vlPFC, ventrolateral prefrontal cortex.

**A. Discovery sample**						**B. Test sample 1**						**C. Test sample 2**					
**Parameter**	**β**	**Hypothesis test**		**Exp (β)**	**Exp(β) 95% CI**	**Parameter**	**β**	**Hypothesis test**		**Exp (β)**	**Exp(β) 95% CI**	**Parameter**	**β**	**Hypothesis test**		**Exp (β)**	**Exp(β) 95% CI**
		**Wald χ^2**	***P* value**					**Wald χ^2**	***P* value**					**Wald χ^2**	***P* value**		
Gender	-0.41	24.33	<.001[a]	0.66	0.56-0.78	Gender	-0.23	9.71	.002[a]	0.79	0.69-0.92	Gender	-0.06	1.68	.194	0.94	0.85-1.03
Age[b]	0.05	38.88	<.001[a]	1.05	1.03-1.07	Age	0.09	185.0	<.001[a]	1.09	1.08-1.10	Age	0.10	134.15	<.001[a]	1.10	1.09-1.12
R vlPFC	-0.59	46.07	<.001[a]	0.55	0.47-0.66	R vlPFC	*N/A*	*N/A*	*N/A*	*N/A*	*N/A*	R vlPFC	*N/A*	*N/A*	*N/A*	*N/A*	*N/A*
R caudate	-0.24	21.00	<.001[a]	0.79	0.71-0.87	R caudate	-0.12	5.55	.018[a]	0.89	0.81-0.98	R caudate	-0.10	3.82	.051	0.91	0.82-1.00
amygdala-L amygdala	0.70	70.87	<.001[a]	2.02	1.71-2.38	amygdala-L amygdala	0.08	0.73	.394	1.08	0.90-1.30	amygdala-L amygdala	0.22	2.25	.133	1.24	0.93-1.65
caudate-L caudate	-0.03	0.09	.762	0.97	0.79-1.19	caudate-L caudate	0.18	6.36	.012[a]	1.20	1.04-1.38	caudate-L caudate	0.02	0.08	.782	1.02	0.89-1.16
dACC-L dlPFC	-0.06	5.67	.017[a]	0.94	0.90-0.99	dACC-L dlPFC	0.03	0.94	.333	1.03	0.97-1.09	dACC-L dlPFC	0.22	7.53	.006[a]	1.25	1.07-1.46
dlPFC-R dlPFC	0.15	16.10	<.001[a]	1.16	1.08-1.25	dlPFC-R dlPFC	0.05	0.58	.447	1.05	0.93-1.18	dlPFC-R dlPFC	-0.99	44.51	<.001[a]	0.37	0.28-0.50
putamen-R OFC ext. to dACC	-0.10	3.72	.054	0.91	0.82-1.00	putamen-R OFC ext. to dACC	*N/A*	*N/A*	*N/A*	*N/A*	*N/A*	putamen-R OFC ext. to dACC	0.43	21.27	<.001[a]	1.54	1.28-1.85
putamen-L dACC	-0.10	3.70	.054	0.91	0.82-1.00	putamen-L dACC	*N/A*	*N/A*	*N/A*	*N/A*	*N/A*	putamen-L dACC	-0.39	12.62	<.001[a]	0.68	0.55-0.84

[a] Raw *P*-values were significant at FDR-corrected thresholds; FDR *q*-values are reported in the eResults.
[b] Age was *z*-scored for the multivariable regression analyses.

© 2023 Schumer MC et al. *JAMA Psychiatry.*

eTable 14. MOODS-SR manic and depressive mood domain correlations with state anxiety, depression severity, and mania/hypomania severity. State anxiety was measured by the Hamilton Anxiety Rating Scale[9]. Depression severity was measured by the Hamilton Rating Scale for Depression[8]. Mania/hypomania severity was measured by the Young Mania Rating Scale[7]. **A.** Discovery sample Pearson correlations. **B.** Test sample 1 Pearson correlations. **C.** Test sample 2 Pearson correlations. Abbreviations: HAMA, Hamilton Anxiety Rating Scale; HRSD, Hamilton Rating Scale for Depression; MOODS-SR, Mood Spectrum Self-Report; YMRS, Young Mania Rating Scale.

**A. Discovery sample (*n*=114)**	**MOODS-SR manic mood domain**	**MOODS-SR depressive mood domain**
**HAMA**	0.487[a]	0.860[a]
**HRSD**	0.462[a]	0.881[a]
**YMRS**	0.556[a]	0.738[a]
**B. Test sample 1 (*n*=103)**	**MOODS-SR manic mood domain**	**MOODS-SR depressive mood domain**
**HAMA[c]**	0.556[a]	0.869[a]
**HRSD**	0.497[a]	0.872[a]
**YMRS**	0.413[a]	0.611[a]
**C. Test sample 2 (*n*=82)**	**MOODS-SR manic mood domain**	**MOODS-SR depressive mood domain**
**HAMA[d]**	0.374[b]	0.468[a]
**HRSD**	0.394[a]	0.525[a]
**YMRS[e]**	0.518[a]	0.337[b]

[a] *P*<.001 (2-tailed).
[b] *P*<.005 (2-tailed).
[c] HAMA score missing for *n*=1 Test sample I participant.
[d] HAMA score missing for *n*=1 Test sample II participant.
[e] YMRS score missing for *n*=1 Test sample II participant.

The correlations among MOODS-SR mania and HRSD and HAMA and among MOODS-SR depression and YMRS and HAMA likely reflect the general aspects of affective psychopathology and arousal, respectively, that are captured by the MOODS-SR mania and depression mood domains.

© 2023 Schumer MC et al. *JAMA Psychiatry.*

eTable 15. Demographic and clinical measures of participants with missing or incomplete data versus participants with complete data from the discovery sample. Age, gender, education, and lifetime psychiatric diagnoses were assessed at time of fMRI scan. Participants may be diagnosed with more than one disorder. Abbreviations: ADHD, Attention Deficit Hyperactivity Disorder; MDD, Major Depressive Disorder; MOODS-SR, Mood Spectrum Self-Report; N/A, not applicable.

**Discovery sample (*n*=133) demographic and clinical variables**	**Participants with complete data (*n*=114)**	**Participants with missing or incomplete data (*n*=19)**	**Statistics**	***P*-value**
Age, mean (SD), years	21.60 (1.91)	22.35 (2.14)	*t*=-1.55	0.123
Gender, No. (%), female	80 (70.18)	11 (58)	χ^2=1.14	0.286
Education, No. (%)			χ^2=0.93	0.629
High school or less	17 (14.91)	4 (21)		
Some college (≥1 year)	67 (58.78)	9 (47)		
Technical school or Associate's degree	0 (0)	0 (0)		
Bachelor's degree or higher	30 (26.31)	6 (32)		
Race, No. (%)			χ^2=2.68	0.444
Asian	18 (15.69)	4 (21)		
Black or African American	12 (10.53)	4 (21)		
White	81 (71.05)	11 (58)		
More than one race	3 (2.63)	0 (0)		
Did not wish to report	0 (0)	0 (0)		
MOODS-SR manic mood domain, mean (SD)	6.94 (5.55)	11.00 (7.94)	*t*=-2.76	0.007
MOODS-SR depressive mood domain, mean (SD)	8.43 (7.89)	10.42 (9.20)	*t*=-0.99	0.322
MDD, No. (%)	28 (24.56)	<10[a]	*N/A*	*N/A*
Any anxiety disorder, No. (%)	28 (24.56)	<10[a]	*N/A*	*N/A*
ADHD, No. (%)	6 (5.26)	<10[a]	*N/A*	*N/A*
Taking psychotropic medication, No. (%)	<10[a]	<10[a]	*N/A*	*N/A*

[a] There were too few participants to provide numbers without compromising identifiability.

© 2023 Schumer MC et al. *JAMA Psychiatry.*

eTable 16. Demographic and clinical measures of participants with missing or incomplete data versus participants with complete data from Test sample 1. Age, gender, education, and lifetime psychiatric diagnoses were assessed at time of fMRI scan. Participants may be diagnosed with more than one disorder. Abbreviations: ADHD, Attention Deficit Hyperactivity Disorder; MDD, Major Depressive Disorder; MOODS-SR, Mood Spectrum Self-Report; N/A, not applicable.

Test sample 1 (*n*=136) demographic and clinical variables	Participants with complete data (*n*=103)	Participants with missing or incomplete data (*n*=33)	Statistics	*P*-value
Age, mean (SD), years	21.57 (2.09)	21.63 (2.15)	t=-0.14	0.887
Gender, No. (%), female	73 (70.87)	25 (76)	χ^2=0.30	0.586
Education, No. (%)			χ^2=0.82	0.844
High school or less	19 (18.45)	7 (21)		
Some college (≥1 year)	53 (51.46)	16 (49)		
Technical school or Associate's degree	2 (1.94)	0 (0)		
Bachelor's degree or higher	29 (28.15)	10 (30)		
Race, No. (%)			χ^2=1.27	0.736
Asian	21 (20.39)	9 (27)		
Black or African American	11 (10.68)	4 (12)		
White	64 (62.13)	19 (58)		
More than one race	7 (6.80)	1 (3)		
Did not wish to report	0 (0)	0 (0)		
MOODS-SR manic mood domain, mean (SD)	7.72 (5.52)	10.15 (5.79)	t=-2.18	0.031
MOODS-SR depressive mood domain, mean (SD)	10.65 (8.67)	13.18 (9.02)	t=-1.44	0.151
MDD, No. (%)	40 (38.84)	14 (42)	χ^2=0.13	0.714
Any anxiety disorder, No. (%)	44 (42.72)	16 (48)	χ^2=0.34	0.562
ADHD, No. (%)	<10[a]	<10[a]	*N/A*	*N/A*
Taking psychotropic medication, No. (%)	<10[a]	<10[a]	*N/A*	*N/A*

[a] There were too few participants to provide numbers without compromising identifiability.

© 2023 Schumer MC et al. *JAMA Psychiatry.*

eTable 17. Demographic and clinical measures of participants with missing or incomplete data versus participants with complete data from Test sample 2. Age, gender, education, and lifetime psychiatric diagnoses were assessed at time of fMRI scan. Participants may be diagnosed with more than one disorder. None of the participants in Test sample II were taking medication. Abbreviations: ADHD, Attention Deficit Hyperactivity Disorder; MDD, Major Depressive Disorder; MOODS-SR, Mood Spectrum Self-Report; N/A, not applicable.

**Test sample 2 (*n*=106) demographic and clinical variables**	**Participants with complete data (*n*=82)**	**Participants with missing or incomplete data (*n*=24)**	**Statistics**	***P*-value**
Age, mean (SD), years	23.43 (2.86)	23.14 (3.24)	t=0.43	0.670
Gender, No. (%), female	48 (58)	15 (62)	χ^2=1.54	0.464
Education, No. (%)			χ^2=2.02	0.567
High school or less	8 (10)	4 (17)		
Some college (≥1 year)	27 (33)	6 (25)		
Technical school or Associate's degree	1 (1)	1 (4)		
Bachelor's degree or higher	46 (56)	13 (54)		
Race, No. (%)			χ^2=2.88	0.577
Asian	17 (21)	2 (8)		
Black or African American	7 (9)	3 (13)		
White	52 (63)	18 (75)		
More than one race	4 (5)	1 (4)		
Did not wish to report	2 (2)	0 (0)		
MOODS-SR manic mood domain, mean (SD)	5.06 (4.97)	6.83 (6.75)	t=-1.41	0.161
MOODS-SR depressive mood domain, mean (SD)	6.27 (7.20)	7.04 (7.21)	t=-0.46	0.645
MDD, No. (%)	11 (13)	<10[a]	*N/A*	*N/A*
Any anxiety disorder, No. (%)	11 (13)	<10[a]	*N/A*	*N/A*
ADHD, No. (%)	<10[a]	0 (0)	*N/A*	*N/A*
Taking psychotropic medication, No. (%)	0 (0)	0 (0)	*N/A*	*N/A*

[a] There were too few participants to provide numbers without compromising identifiability.

© 2023 Schumer MC et al. *JAMA Psychiatry.*

eTable 18. Family psychiatric history information for the three samples. Number of participants with a family history of Bipolar Disorder, Major Depressive Disorder, Attention Deficit Hyperactivity Disorder, or anxiety disorder.

Family Psychiatric History[a], No. (%)	Discovery sample (*n*=114)	Test sample 1 (*n*=103)	Test sample 2 (*n*=82)	Statistics	*P* value
Bipolar Disorder	2 (1.75)	7 (6.80)	2 (2.44)	χ^2=4.37	.112
Major Depressive Disorder	20 (17.54)	27 (26.21)	11 (13.41)	χ^2=5.19	.075
Attention Deficit Hyperactivity Disorder	6 (5.26)	4 (3.88)	2 (2.44)	χ^2=0.99	.608
Anxiety Disorder	13 (11.40)	16 (15.54)	11 (13.41)	χ^2=0.80	.671

[a] First-degree relatives only.

© 2023 Schumer MC et al. *JAMA Psychiatry.*

eReferences

1. First M, Williams J, Karg R, Spitzer R. *Structured Clinical Interview for DSM-5—Research Version (SCID-5 for DSM-5, Research Version; SCID-5-RV).* American Psychiatric Association; 2015.

2. Fagiolini A, Dell'osso L, Pini S, et al. Validity and reliability of a new instrument for assessing mood symptomatology: the Structured Clinical Interview for Mood Spectrum (SCI-MOODS). *International Journal of Methods in Psychiatric Research*. 1999;8(2):71-82. doi:10.1002/mpr.58

3. Cassano GB, Mula M, Rucci P, et al. The structure of lifetime manic–hypomanic spectrum. *Journal of Affective Disorders*. 2009;112(1):59-70. doi:10.1016/j.jad.2008.04.019

4. Dell'Osso L, Armani A, Rucci P, et al. Measuring mood spectrum: Comparison of interview (SCI-MOODS) and self-report (MOODS-SR) instruments. *Comprehensive Psychiatry*. 2002;43(1):69-73. doi:10.1053/comp.2002.29852

5. Cassano GB, Rucci P, Frank E, et al. The Mood Spectrum in Unipolar and Bipolar Disorder: Arguments for a Unitary Approach. *AJP*. 2004;161(7):1264-1269. doi:10.1176/appi.ajp.161.7.1264

6. Cassano GB, Dell'Osso L, Frank E, et al. The bipolar spectrum: a clinical reality in search of diagnostic criteria and an assessment methodology. *Journal of Affective Disorders*. 1999;54(3):319-328. doi:10.1016/S0165-0327(98)00158-X

7. Young RC, Biggs JT, Ziegler VE, Meyer DA. A Rating Scale for Mania: Reliability, Validity and Sensitivity. *The British Journal of Psychiatry*. 1978;133(5):429-435. doi:10.1192/bjp.133.5.429

8. Hamilton M. A RATING SCALE FOR DEPRESSION. *J Neurol Neurosurg Psychiatry*. 1960;23(1):56-62.

9. Hamilton M. The assessment of anxiety states by rating. *British Journal of Medical Psychology*. 1959;32:50-55. doi:10.1111/j.2044-8341.1959.tb00467.x

10. Smith SM, Jenkinson M, Woolrich MW, et al. Advances in functional and structural MR image analysis and implementation as FSL. *NeuroImage*. 2004;23:S208-S219. doi:10.1016/j.neuroimage.2004.07.051

11. Jenkinson M, Beckmann CF, Behrens TEJ, Woolrich MW, Smith SM. FSL. *NeuroImage*. 2012;62(2):782-790. doi:10.1016/j.neuroimage.2011.09.015

12. Cox RW. AFNI: Software for Analysis and Visualization of Functional Magnetic Resonance Neuroimages. *Computers and Biomedical Research*. 1996;29(3):162-173. doi:10.1006/cbmr.1996.0014

13. Gorgolewski K, Burns C, Madison C, et al. Nipype: A Flexible, Lightweight and Extensible Neuroimaging Data Processing Framework in Python. *Frontiers in Neuroinformatics*. 2011;5. Accessed June 17, 2022. https://www.frontiersin.org/article/10.3389/fninf.2011.00013

14. Esteban O, Markiewicz CJ, Blair RW, et al. fMRIPrep: a robust preprocessing pipeline for functional MRI. *Nat Methods*. 2019;16(1):111-116. doi:10.1038/s41592-018-0235-4

15. Kundu P, Voon V, Balchandani P, Lombardo MV, Poser BA, Bandettini PA. Multi-echo fMRI: A review of applications in fMRI denoising and analysis of BOLD signals. *NeuroImage*. 2017;154:59-80. doi:10.1016/j.neuroimage.2017.03.033

16. Kundu P, Inati SJ, Evans JW, Luh WM, Bandettini PA. Differentiating BOLD and non-BOLD signals in fMRI time series using multi-echo EPI. *NeuroImage*. 2012;60(3):1759-1770. doi:10.1016/j.neuroimage.2011.12.028

© 2023 Schumer MC et al. *JAMA Psychiatry.*

17. DuPre E, Salo T, Ahmed Z, et al. TE-dependent analysis of multi-echo fMRI with *tedana*. *Journal of Open Source Software*. 2021;6(66):3669. doi:10.21105/joss.03669

18. Fournier JC, Keener MT, Almeida J, Kronhaus DM, Phillips ML. Amygdala and whole-brain activity to emotional faces distinguishes major depressive disorder and bipolar disorder. *Bipolar Disorders*. 2013;15(7):741-752. doi:10.1111/bdi.12106

19. Steele JS, Bertocci M, Eckstrand K, et al. A specific neural substrate predicting current and future impulsivity in young adults. *Mol Psychiatry*. 2021;26(9):4919-4930. doi:10.1038/s41380-021-01017-0

20. Perlman SB, Almeida JR, Kronhaus DM, et al. Amygdala activity and prefrontal cortex–amygdala effective connectivity to emerging emotional faces distinguish remitted and depressed mood states in bipolar disorder. *Bipolar Disord*. 2012;14(2):162-174. doi:10.1111/j.1399-5618.2012.00999.x

21. Acuff HE, Versace A, Bertocci MA, et al. Association of Neuroimaging Measures of Emotion Processing and Regulation Neural Circuitries With Symptoms of Bipolar Disorder in Offspring at Risk for Bipolar Disorder. *JAMA Psychiatry*. 2018;75(12):1241-1251. doi:10.1001/jamapsychiatry.2018.2318

22. Tottenham N, Tanaka JW, Leon AC, et al. The NimStim set of facial expressions: Judgments from untrained research participants. *Psychiatry Research*. 2009;168(3):242-249. doi:10.1016/j.psychres.2008.05.006

23. Maldjian JA, Laurienti PJ, Kraft RA, Burdette JH. An automated method for neuroanatomic and cytoarchitectonic atlas-based interrogation of fMRI data sets. *NeuroImage*. 2003;19(3):1233-1239. doi:10.1016/S1053-8119(03)00169-1

24. Picard RR, Cook RD. Cross-Validation of Regression Models. *Journal of the American Statistical Association*. 1984;79(387):575-583. doi:10.1080/01621459.1984.10478083

© 2023 Schumer MC et al. *JAMA Psychiatry.*
