# Supplementary material for: Patterns of Neural Network Functional Connectivity Associated With Mania/Hypomania and Depression Risk in 3 Independent Young Adult Samples
Source: JAMA Psychiatry. 2023 Nov 1;81(2):167–77. doi: 10.1001/jamapsychiatry.2023.4150 (PMC10620679; doi:10.1001/jamapsychiatry.2023.4150)
Supplement: Supplement 2. — Data sharing statement [file jamapsychiatry-e234150-s002.pdf]

Data Sharing Statement

Schumer. Patterns of Neural Network Functional Connectivity Associated With Mania/Hypomania and Depression Risk in 3 Independent Young Adult Samples. *JAMA Psychiatry*. Published November 01, 2023. doi:10.1001/jamapsychiatry.2023.4150

Data
Data available: Yes
Data types: Deidentified participant data, Data dictionary
How to access data: NIMH Data Archive (NDA) repository NIMH Data Archive - Data - Collection (nih.gov) Collection title: Reward, impulsive sensation seeking and emotional dysregulation: neural mechanisms underlying risk for bipolar disorder in young adults URL: https://nda.nih.gov/edit_collection.html?id=3397
When available: With publication

Supporting Documents
Document types: None

Additional Information
Who can access the data: Supporting documents are in the public domain.
Types of analyses: Please see above.
Mechanisms of data availability: Data is in the public domain/NDA archive specified above.
